# Substance use risk among school going adolescents in India: secondary analysis of Community Based Peer-Led Intervention (CPLI) trial

**DOI:** 10.1016/j.lansea.2026.100821

**Published:** 2026-07-22

**Authors:** U. Venkatesh, Mahashweta Chakrabarty, Palanivel Chinnakali, Krupal Joshi, Sudip Bhattacharya, Aravind P. Gandhi, Rashmi Agarwalla, Richa Tripathi, Vinoth Rajendran, Anand Mohan Dixit

**Affiliations:** a Department of Community and Family Medicine, All India Institute of Medical Sciences Gorakhpur, Uttar Pradesh, India; b Department of Preventive and Social Medicine, Jawaharlal Institute of Postgraduate Medical Education and Research, Puducherry, India; c Department of Community and Family Medicine, All India Institute of Medical Science -Rajkot, Gujarat, India; d Department of Community and Family Medicine, All India Institute of Medical Science, Deoghar, Jharkhand, India; e Department of Community Medicine, All India Institute of Medical Sciences, Nagpur, Maharashtra, India; f Department of Community and Family Medicine, All India Institute of Medical Sciences, Guwahati, India; g Department of Psychiatry, All India Institute of Medical Sciences Gorakhpur, India

**Keywords:** Adolescents, Substance use, ASSIST, Regional inequality, Fairlie decomposition, India, School Health Programme, Ayushman Bharat

## Abstract

**Background:**

Adolescent substance use is an increasing public health concern in India, yet multicentre studies are limited. In this study we aimed to understand variation in substance use risk across multiple schools in selected districts of India.

**Methods:**

This study utilised cross-sectional baseline data from a multi-centre cluster randomised trial on peer-led intervention to reduce substance use, it included 6168 adolescents from 108 schools across six Indian districts (2025–2026). Substance use risk was assessed through self-reporting of participants using the WHO Alcohol, Smoking and Substance Involvement Screening Test and personality traits were assessed using the Substance Use Risk Profile Scale (SURPS). Determinants were examined using three-level multilevel ordinal logistic regression models accounting for clustering at school levels. Inter-district disparities were investigated using Fairlie decomposition.

**Findings:**

Highest substance use risk was observed in selected schools of Guwahati and lowest in Nagpur and Puducherry. Higher accessibility to substances (high: aOR 1.75, 95% CI: 1.36–2.26), high digital exposure (aOR 1.44, 95% CI: 1.16–1.79), and favourable attitudes towards substance use (aOR 1.30, 95% CI: 1.01–1.69) were associated with increased risk, whereas male adolescents had lower odds than females (aOR 0.74, 95% CI: 0.62–0.88). The Guwahati–Nagpur prevalence gap was 35.1 percentage points, of which 37.9% was explained by observed characteristics, mainly school characteristics, socio-demographic and economic factors, and personality traits.

**Interpretation:**

Substance use risk among adolescents in India varies substantially across settings. Context-specific strategies targeting digital exposure, and local environments may help reduce inequalities in substance use risk.

**Funding:**

This study is supported by Indian Council of Medical Research, Extramural Research Grant (2024-01-00480).

**Research in context**

**Evidence before this study**

We searched PubMed using the terms “adolescent substance use”, “India”, “school-based”, “risk factors”, “ASSIST”, “regional variation”, and “inequality” for studies published between January 1, 2000, and December 31, 2025. Existing evidence from India largely focuses on prevalence, specific substances (such as tobacco or alcohol), or determinants within single geographic settings. Some national and subnational studies have identified socio-demographic and behavioural correlates of substance use among adolescents; however, few studies have examined multi-substance risk using standardised tools such as the World Health Organisation (WHO) Alcohol, Smoking and Substance Involvement Screening Test (ASSIST) tool. Importantly, there is limited evidence on how substance use risk varies across diverse settings in India and the extent to which observed differences are accounted for by measurable characteristics versus broader contextual and structural factors. Studies exploring inequalities using decomposition-based approaches in adolescent substance use are particularly scarce.

**Added value of this study**

This study provides multi-site self-reported adolescent substance use risk in India using a standardised risk classification (WHO ASSIST) in selected schools across six districts. By combining multivariable logistic regression with Fairlie decomposition, the study goes beyond identifying determinants to examining factors associated with observed setting-specific disparities. The findings demonstrate substantial inter-site heterogeneity, with markedly higher risk in selected schools belonging to Guwahati and Deoghar compared to Puducherry and Nagpur. Key modifiable determinants associated with higher substance use risk included accessibility to substances, digital exposure, and risky behavioural environments, while social engagement shows a protective effect. The decomposition analysis reveals that only a modest proportion of inter-site differences was accounted for by measured characteristics, suggesting that additional contextual and structural factors may contribute to the observed disparities. The study also demonstrates robust model performance, strengthening the validity of these findings.

**Implications of all the available evidence**

The findings suggest that adolescent substance use risk in India is driven by contextual environments. This has important implications for policy and programme design. Prevention strategies may benefit from moving beyond generic awareness-based approaches to context-specific, multi-level interventions. Strengthening regulatory enforcement to reduce accessibility, addressing digital exposure through media literacy, and scaling peer-led models such as the Community Based Peer-Led Intervention (CPLI) within the School Health Programme may support prevention efforts, although their effectiveness requires further evaluation. Integrating these approaches within existing school and primary healthcare platforms may facilitate early identification and prevention. Overall, addressing adolescent substance use risk may require coordinated efforts informed by setting-specific differences and local contextual factors.

## Introduction

Adolescence represents a critical developmental period marked by rapid physical, psychological, and social transitions, during which behavioural patterns are established that can have long-term consequences for health and well-being.^1^ Risk-taking behaviours initiated during this stage, including substance use, often persist into adulthood and may influence not only individual life trajectories but also broader intergenerational health outcomes.^2^^,^^3^ In India, recent evidence indicates a wide range of substance use, including tobacco, alcohol, cannabis, inhalants, and pharmaceutical drugs, which are linked to increased risk of addiction, mental health disorders, poor academic performance, school dropout, and engagement in other high-risk behaviours.^4^, ^5^, ^6^, ^7^, ^8^, ^9^, ^10^ These trends underscore the urgency of understanding not only the prevalence of substance use among adolescents, but also how associated risks are distributed across different contexts.

In response, national initiatives such as the School Health Programme under Ayushman Bharat have prioritised adolescent health, including substance use prevention through school-based strategies.^11^ However, existing approaches have largely relied on awareness-driven and didactic models, which have shown limited effectiveness in achieving sustained behavioural change.^12^ This has led to growing interest in more context-sensitive approaches, including Community Based Peer-Led Interventions (CPLI), which are better aligned with adolescent social environments and behavioural dynamics.

A growing body of evidence suggests that adolescent substance use is influenced by a complex interplay of individual, social, and environmental factors. Previous studies have identified substance-related attitudes, accessibility to substances, digital media exposure, social influences, behavioural risk environments, and personality traits such as hopelessness, anxiety sensitivity, impulsivity, and sensation seeking as important determinants of substance use behaviours and vulnerability.^13^, ^14^, ^15^, ^16^, ^17^ These factors may function as either risk or protective influences and therefore were included in the present study to provide a comprehensive assessment of substance use risk among adolescents.

Despite increasing research on adolescent substance use in India, important gaps remain. First, most existing studies are limited to single substances or specific geographic locations, restricting understanding of broader patterns across diverse contexts.^18^, ^19^, ^20^, ^21^, ^22^, ^23^, ^24^ Second, there is limited evidence on the combined burden of multiple substance use and the classification of adolescents into risk categories using standardised tools such as the World Health Organisation (WHO) Alcohol, Smoking and Substance Involvement Screening Test (ASSIST) tool.^13^^,^^18^^,^^25^ Existing studies have largely focused on identifying individual-level correlates of substance use, with limited attention to quantifying the contribution of different domains of factors to observed regional disparities.^18^^,^^23^^,^^24^ Decomposition-based approaches, which can distinguish the contribution of measurable characteristics from unexplained contextual influences, remain rarely applied in adolescent substance use research in India.^13^^,^^26^ In particular, little is known about the extent to which observed differences are driven by measurable characteristics versus unobserved contextual influences.

The present study aims to examine substance use among school-going adolescents across these study sites by assessing patterns of use across multiple substances; estimating the prevalence of moderate-to-high risk using the WHO ASSIST tool; identifying key determinants of elevated risk; and quantifying inequalities in substance use risk and decomposing these differences to identify contributing factors.

## Methods

This study utilised cross-sectional baseline data from a multi-centre project titled *“Evaluating the Effectiveness of a Community* *Based Peer-Led Intervention (CPLI) in Preventing Substance Use among Adolescents: A Cluster Randomised Controlled Trial within the School Health Programme under Ayushman Bharat.”* The baseline assessment, conducted prior to intervention implementation, forms the basis of this analysis. While the parent study is a cluster randomised controlled trial (registration number: CTRI/2025/04/083912), the present analysis is cross-sectional and followed STROBE guidelines.^27^

The study was conducted across six district-level sites in India, Gorakhpur (Uttar Pradesh), Kamrup/Guwahati (Assam), Nagpur (Maharashtra), Rajkot (Gujarat), Deoghar (Jharkhand), and Puducherry, capturing urban, semi-urban, rural, and tribal school settings. The geographical distribution of the study sites is presented in Supplementary Figure S1. Data collection occurred between October 2025 and January 2026, coordinated by All India Institute of Medical Sciences (AIIMS) Gorakhpur, AIIMS Guwahati, AIIMS Nagpur, AIIMS Rajkot, AIIMS Deoghar, and JIPMER Puducherry.

The study included school-going adolescents between 10 and 19 years of age from government and private schools across the six study sites. Eligible participants were students enrolled in the selected schools, present on the day of data collection, and willing to provide assent along with parental/guardian consent for those younger than 18 years. Students who were absent during data collection, declined participation, provided incomplete questionnaires, or were unable to respond due to cognitive or communication limitations were excluded. Within each participating school, one class was randomly selected for survey administration. To ensure adequate participant numbers within the sampled classroom, only classes with at least 50 enrolled students were considered eligible for selection. This criterion related to class size and not to student participation rates.

Schools were required to provide institutional permission for participation. Schools currently involved in substance use-related research studies or intervention projects similar to the present study were excluded to minimise potential research-related bias arising from prior exposure to comparable activities.

The present study utilised baseline data collected as part of a larger multi-site trial conducted across six study sites in India. Sample size estimation for the parent trial was performed using G∗Power (version 3.1.9.4), assuming a two-sided significance level (α = 0.05), 80% power, and a moderate effect size (Cohen's d = 0.5). Given the cluster-based study design, a design effect was incorporated to account for intra-cluster correlation (ICC = 0.05).

In the parent trial, 18 schools were selected from each study site, comprising 9 schools allocated to the intervention arm and 9 schools allocated to the control arm. Thus, across the six study sites, a total of 108 schools were included (18 schools × 6 sites = 108 schools; 54 intervention schools and 54 control schools). Schools in the parent trial were subsequently randomised in a 1:1 ratio using stratified block randomisation within each study site.

**Fig. 1:**
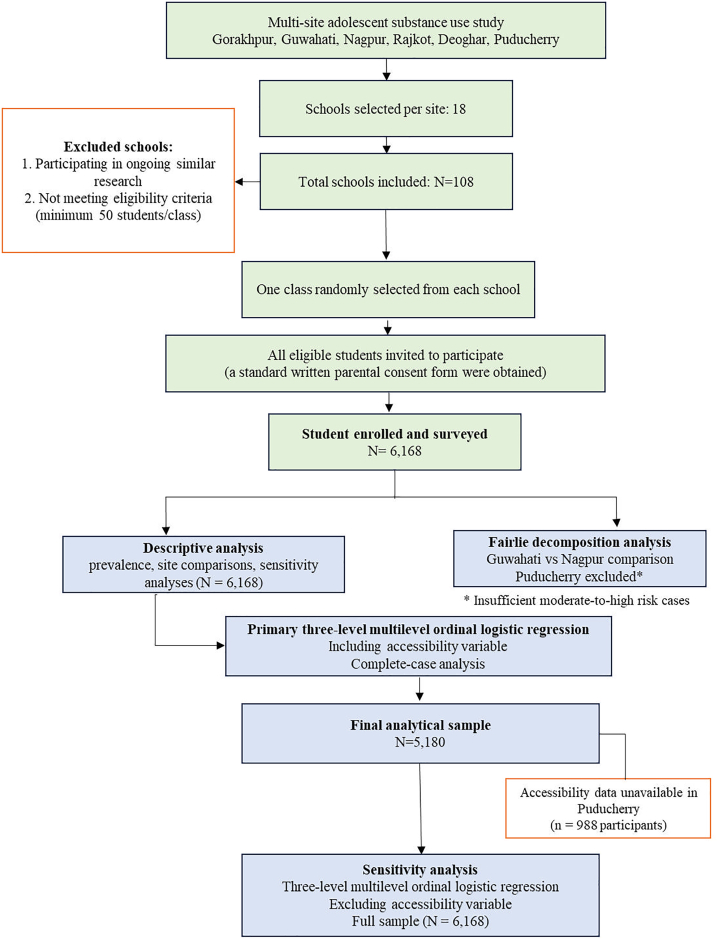
**Flowchart showing study participant selection and analytical samples used for descriptive, multilevel regression, sensitivity, and decomposition analyses**.

For the present baseline analysis, a multistage cluster sampling approach was used, with schools serving as the primary sampling units. Within each study site, schools were stratified by location (urban/rural) and management type (government/private) and selected using computer-generated random sampling procedures. Within each selected school, one eligible class was randomly selected, and all students present on the day of data collection who met the eligibility criteria were invited to participate. A total of 6168 adolescents participated in the baseline survey. The site-wise distribution of participants was as follows: Gorakhpur (n = 1223), Guwahati (n = 890), Nagpur (n = 1082), Rajkot (n = 919), Deoghar (n = 1066), and Puducherry (n = 988). A participant flowchart illustrating the selection of study sites, schools, classes, and participants, as well as the final analytical samples used in the analyses, has been provided in Fig. 1.

Data were collected using a structured, self-administered questionnaire covering substance use, digital exposure, social engagement, psychosocial characteristics, and socio-demographic factors. The questionnaire was administered in a bilingual format comprising English and the respective local language of each study site. Prior to field implementation, the questionnaire underwent face validation by subject experts and was pilot tested at all study sites in schools with similar socio-demographic characteristics that were not included in the main study. Feedback from the pilot exercise was used to refine questionnaire wording, improve comprehension, and standardise administration procedures. Before questionnaire administration, trained research staff conducted a standardised orientation session using PowerPoint presentations to explain the study objectives, questionnaire items, and procedures for completing the questionnaire. Data collection was conducted in an examination-like setting to facilitate independent responses and ensure confidentiality. Students completed the questionnaires themselves, while trained field investigators remained present to clarify queries when required but did not influence, guide, or record participant responses. To maintain privacy, students were seated with adequate spacing and were instructed not to discuss their responses during questionnaire completion. Completed questionnaires were submitted anonymously and were not accessible to teachers or school staff. School personnel were involved only in logistical coordination and classroom organisation and had no role in questionnaire administration, response recording, or data management. All data collectors received standardised training before field implementation to ensure uniform procedures across study sites. Within each selected school, one class was randomly selected, and all eligible students present in that class on the day of data collection were invited to participate. Data collection was supervised using standardised protocols to ensure consistency across study sites and minimise potential sources of bias.

Substance use risk was assessed using the WHO-ASSIST version 3.1.^28^ The ASSIST instrument evaluates substance-specific involvement across multiple behavioural and consequence-related domains, including frequency of use, craving or strong desire to use, health, social or legal problems related to use, failure to fulfil expected roles or responsibilities, concern expressed by family members or others, and unsuccessful attempts to reduce or control use. These domains collectively reflect both the intensity of use and its associated adverse consequences.

For each substance, a composite ASSIST score was calculated by summing weighted responses across domains. Based on WHO-recommended cut-offs, scores were categorised into low, moderate, and high risk. For tobacco and other substances, scores of 0–3 were classified as low risk, 4–26 as moderate risk, and ≥27 as high risk; for alcohol, scores of 0–10 were considered low risk, 11–26 as moderate risk, and ≥27 as high risk.^28^

For the primary multilevel analysis, a three-category ordinal variable (low risk, moderate risk, and high risk) was used as the outcome in the three-level multilevel ordinal logistic regression analysis. To derive an overall substance use risk measure, participants were classified according to the highest ASSIST risk category reported across all assessed substances. Adolescents were classified as low risk if they had low-risk ASSIST scores for all substances. They were classified as moderate risk if they had a moderate-risk ASSIST score for at least one substance and no high-risk score for any substance. Adolescents were classified as high risk if they had a high-risk ASSIST score for at least one substance, irrespective of their risk levels for other substances. For the Fairlie decomposition analysis, the outcome was further recoded into a binary variable, where adolescents classified as moderate risk or high risk were grouped as having moderate/high substance use risk (coded as 1) and those classified as low risk across all substances were grouped as low risk (coded as 0).

**Fig. 2:**
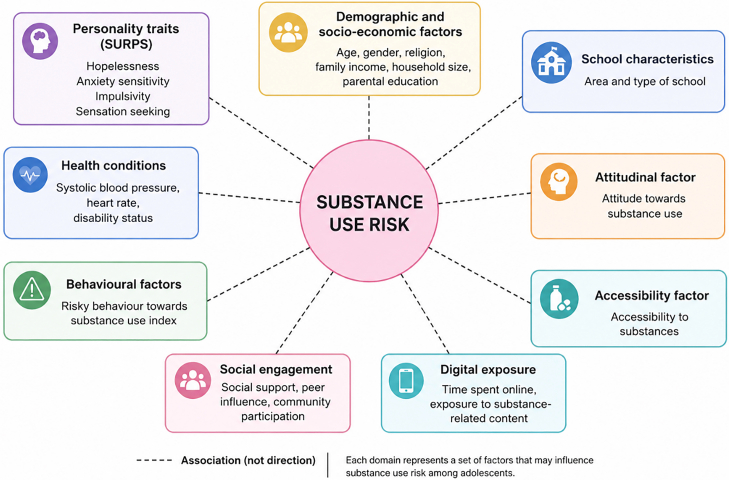
**Conceptual framework illustrating the determinants of moderate-to-high substance use risk among school-going adolescents**.

The selection of independent variables was guided by existing literature on adolescent substance use and behavioural risk factors.^13^^,^^29^, ^30^, ^31^, ^32^ Variables were grouped into key domains, including socio-demographic and economic characteristics, school characteristics, attitudinal factors, accessibility to substances, digital exposure, social engagement, risky behavioural environment, health-related factors like disability, blood pressure, and heart rate, and personality traits (see Fig. 2).

Several constructs, including personality traits (hopelessness, anxiety sensitivity, impulsivity, and sensation seeking), attitudes towards substance use, accessibility, digital exposure, social engagement, and risky behavioural environment, were measured using composite indices derived from multiple items. Detailed definitions, item construction, and categorisation of all variables are provided in Supplementary Table S1.

### Statistical analysis

Continuous variables were presented as means and standard deviations (SD), while categorical variables were summarised as frequencies and percentages. Site-wise differences were assessed using one-way analysis of variance (ANOVA) for continuous variables and Pearson's chi-square test for categorical variables. Post-hoc pairwise comparisons were conducted using Bonferroni-adjusted tests to identify specific inter-site differences in substance use risk (Supplementary Table S3).

Composite indices, including anti-substance use attitude, digital exposure, social engagement, risky behaviour towards substance use, and the four Substance Use Risk Profile Scale (SURPS) personality domains, were constructed from multiple questionnaire items. Internal consistency was assessed using Cronbach's alpha coefficients (Supplementary Table S2).

To address the objective of identifying determinants of substance use risk, three-level multilevel ordinal logistic regression models were fitted with students nested within schools and schools nested within study sites. The outcome variable comprised three ordered categories of substance use risk (low, moderate, and high). Prior to model estimation, an unconditional (null) three-level ordinal logistic regression model with random intercepts at the school and study-site levels was estimated to assess clustering. The likelihood-ratio test comparing the multilevel model with a conventional ordinal logistic regression model confirmed statistically significant clustering, supporting the use of a multilevel modelling framework (Supplementary Table S5).

Before multivariable modelling, multicollinearity among explanatory variables was assessed using Variance Inflation Factors (VIF). All VIF values, including the mean VIF, were below 5, indicating no evidence of problematic multicollinearity (Supplementary Table S4). Adjusted odds ratios (aORs), 95% confidence intervals (CIs), and p-values were reported, with statistical significance assessed at p < 0.05. Model fit was evaluated using the log-likelihood, Akaike Information Criterion (AIC), Bayesian Information Criterion (BIC), and likelihood-ratio (LR) test (Supplementary Table S5).

For multivariable modelling, complete-case analysis was employed, whereby observations with missing values on variables included in the model were excluded during estimation. Because accessibility-related information was unavailable for all participants from the Puducherry study site (n = 988), the primary multilevel model that included accessibility as a covariate was estimated on an analytic sample of 5180 adolescents. To assess the robustness of the findings, a sensitivity analysis was conducted by re-estimating the model after excluding the accessibility variable, thereby allowing inclusion of the full study sample (N = 6168) (Supplementary Table S19). In a second sensitivity analysis, the four SURPS personality domains (hopelessness, anxiety sensitivity, impulsivity, and sensation seeking) were categorised into tertiles (low, moderate, and high) and re-entered into the multilevel model to examine whether findings were sensitive to alternative specifications of personality traits (Supplementary Table S6).

As an additional analysis, substance-specific three-level multilevel ordinal logistic regression models were estimated separately for tobacco, alcohol, cannabis, cocaine, amphetamines, inhalants, sedatives, hallucinogens, and opioids to examine whether determinants differed across substance categories (Supplementary Tables S7–S15).

To address the objective of examining inequalities in substance use risk across study sites and identifying factors contributing to these disparities, Fairlie decomposition analysis was performed. Fairlie decomposition is an extension of the Blinder–Oaxaca decomposition suitable for binary outcomes and quantifies the extent to which differences in observed characteristics explain differences in outcome prevalence between groups.^33^

The decomposition analysis was performed between Guwahati, which exhibited the highest prevalence of moderate-to-high risk, and Nagpur, which had the lowest prevalence among sites with sufficient cases. Although Puducherry showed the lowest prevalence overall, it was excluded from the decomposition analysis due to an insufficient number of respondents reporting moderate-to-high risk, which limited meaningful comparative analysis.

The Fairlie decomposition was implemented using the “*fairlie*” command in Stata, which allows decomposition of differences in binary outcomes based on nonlinear models. All statistical analyses were performed using Stata 16 MP statistical software.^34^

### Ethics statement

The study received ethical approval from the Institutional Human Ethics Committees of AIIMS Gorakhpur (Ref No: IHEC/AIIMS-GKP/BMR/440/2024), AIIMS Guwahati (Ref No. AIIMSG/IEC/M7/F210/2024), AIIMS Nagpur (Ref No. IEC/Pharmac/2025/1224), AIIMS Deoghar (Ref No. AIIMS-DEO/RC-IEC-Subcommittee/2025/1962), AIIMS Rajkot (Ref No. AIIMS/RAJKOT/6th IEC/FB/37), and JIPMER Pondicherry (Ref No. JIP/IECIS-1/0125/17). Administrative permissions were obtained from district education authorities and participating schools. Informed consent was obtained from all adolescents, and written parental/guardian consent was secured for participants below 18 years. Participation was voluntary, and confidentiality of responses was strictly maintained throughout the study.

### Role of the funding source

The funding agency had no role in the study design; data collection, analysis, or interpretation; manuscript writing; or the decision to submit the article for publication.

## Results

Differences in socio-demographic and behavioural characteristics were observed across the six selected study sites (Table 1). Age distribution differed notably across sites, with respondents in Nagpur being younger (mean, standard deviation: 12.4 ± 1.4 years) compared to other sites, while Gorakhpur had the oldest cohort (15.6 ± 1.3 years). Overall, 50.7% of participants were male, although females predominated in Guwahati, Nagpur, Rajkot, and Deoghar.

**Table 1:** Regional variation in distribution of socio-demographic, economic, behavioural, and substance use-related characteristics of adolescent school children across six study-sites.

Variables	Gorakhpur (n = 1223)	Guwahati (n = 890)	Nagpur (n = 1082)	Rajkot (n = 919)	Deoghar (n = 1066)	Puducherry (n = 988)	p value
	Mean ± SD						
Age	15.56 ± 1.34	15.08 ± 1.40	12.42 ± 1.42	14.74 ± 1.16	13.86 ± 1.60	14.72 ± 1.10	<0.001
Number of household members (mean)	6 ± 3	4 ± 2	5 ± 2	6 ± 2	6 ± 3	5 ± 1	<0.001
Anti-substance use attitude index (mean)	4.28 ± 0.86	3.75 ± 0.65	4.32 ± 1.01	4.31 ± 0.72	3.95 ± 0.95	4.98 ± 0.15	<0.001
Accessibility of substances index (mean)	1.74 ± 1.08	3.01 ± 1.29	2.29 ± 1.12	1.62 ± 0.87	1.76 ± 1.02	a	<0.001
Social engagement index (mean)	3.36 ± 1.08	3.35 ± 0.89	3.44 ± 1.07	3.52 ± 0.9	3.31 ± 1.01	3.21 ± 0.87	<0.001
Digital exposure index (mean)	1.87 ± 0.71	2.92 ± 0.73	1.72 ± 0.82	2.1 ± 0.83	1.85 ± 0.75	2.08 ± 0.73	<0.001
Risky behaviour towards substance use index score	21.98 ± 6.61	26.08 ± 8.88	20.82 ± 6.76	24.2 ± 6.51	23.13 ± 6.34	22.6 ± 5.88	<0.001
Personality traits							
Hopelessness	11.32 ± 4.64	16.70 ± 4.39	10.29 ± 5.56	12.59 ± 4.69	13.35 ± 5.19	23.1 ± 8.37	<0.001
Anxiety Sensitivity	15.16 ± 5.38	16.57 ± 4.14	14.46 ± 6.57	14.92 ± 4.68	14.68 ± 5.3	15.31 ± 4.34	<0.001
Impulsivity	14.61 ± 4.73	16.78 ± 3.8	12.66 ± 5.86	14.12 ± 4.3	14.06 ± 4.87	15.42 ± 4.01	<0.001
Sensation Seeking	19.05 ± 5.68	20.17 ± 4.61	16.39 ± 6.75	19.4 ± 4.73	17.65 ± 5.82	20.72 ± 4.86	<0.001
Percentage							
Gender							<0.001
Female	40.07	59.21	53.51	54.30	52.53	40.69	
Male	59.93	40.79	46.49	45.70	47.47	59.31	
Monthly family income							<0.001
Low income	51.1	26.29	54.16	27.86	46.25	25.4	
Middle income	26.82	34.38	25.69	36.89	38.09	27.53	
High income	22.08	39.33	20.15	35.26	15.67	47.06	
Father's education							<0.001
Low	40.39	40.45	62.29	89.88	58.91	27.63	
Secondary	48.16	51.46	33.64	8.05	36.59	53.04	
Higher	11.45	8.09	4.07	2.07	4.5	19.33	
Mother's education							<0.001
Low	57.4	43.82	62.2	94.12	71.29	29.45	
Secondary	35.49	52.81	33.73	4.46	25.33	49.29	
Higher	7.11	3.37	4.07	1.41	3.38	21.26	
Religion							<0.001
Hindu	93.46	69.1	80.22	91.08	86.87	88.97	
Muslim	6.21	30.45	7.49	8.16	12.1	3.95	
Others	0.33	0.45	12.29	0.76	1.03	7.09	
Disability							<0.001
No	89.21	96.85	94.64	96.3	85.74	98.68	
Yes	10.79	3.15	5.36	3.7	14.26	1.32	
Area of school							<0.001
Rural	38.51	50.56	47.69	50.38	60.79	39.07	
Urban	61.49	49.44	52.31	49.62	39.21	60.93	
Type of school							<0.001
Public	51.27	82.25	99.35	100	100	49.39	
Private	48.73	17.75	0.65	0	0	50.61	

For descriptive purposes, raw scores of personality traits are presented in this table.

a Accessibility of substances Index was not available for Puducherry because of non-response in the accessibility-of substances section; therefore, a site-specific mean score could not be calculated.

Adolescents belonging to selected schools from Guwahati reported the highest mean digital exposure score (2.9 ± 0.7) and risky behavioural environment score (26.1 ± 8.9), whereas those from schools belonging to Nagpur and Rajkot generally reported lower levels of these factors. Conversely, anti-substance use attitudes were strongest in selected schools of Puducherry (5.0 ± 0.2) and weakest in Guwahati (3.8 ± 0.7). Social engagement varied relatively little across sites (Table 1).

Overall, 21.4% of adolescents were reported as having moderate/high substance use risk, including 10.3% with moderate risk and 11.1% with high risk. The remaining adolescents (78.6%) were reported as low risk. There was a statistically significant variation in substance use risk across the six study sites (Pearson χ^2^ (10) = 1200.0, p < 0.001) (Table 2). Schools selected from Guwahati and Deoghar had the highest proportion of adolescents reported to be in the high-risk and moderate-risk categories. In contrast, selected schools belonging to Nagpur and Puducherry were characterised by predominantly low-risk profiles (Table 2).

**Table 2:** Regional variation in substance use risk across the six study sites (N = 6168).

Study site	Low risk n (%)	Moderate risk n (%)	High risk n (%)	Moderate/High risk n (%)
Gorakhpur	923 (75.47)	183 (14.96)	117 (9.57)	300 (24.53)
Guwahati	514 (57.75)	199 (22.36)	177 (19.89)	376 (42.25)
Nagpur	1005 (92.88)	31 (2.87)	46 (4.25)	77 (7.12)
Rajkot	795 (86.51)	109 (11.86)	15 (1.63)	124 (13.49)
Deoghar	626 (58.72)	109 (10.23)	331 (31.05)	440 (41.28)
Puducherry	987 (99.90)	1 (0.10)	0 (0.00)	1 (0.10)

χ^2^ test for three-category risk distribution: Pearson χ^2^ (10) = 1200.0, *p* < 0.001.

χ^2^ test for moderate/high risk prevalence: Pearson χ^2^ (5) = 920.29, *p* < 0.001.

Percentages are column percentages within each study site. Moderate/high risk was defined as reporting either moderate-risk or high-risk substance use according to the ASSIST risk classification.

When moderate- and high-risk categories were combined, the prevalence of elevated substance use risk varied across the sites ranging from 42.3% in Guwahati to 7.1% in Nagpur and 0.1% in Puducherry. Post-hoc pairwise comparisons with Bonferroni correction showed that selected schools belonging to both Guwahati and Deoghar sites had higher prevalence of moderate/high substance use risk than Nagpur and Rajkot (Supplementary Table S3). These findings require cautious interpretation considering the self-reported nature of the substance use risk.

The largest meaningful disparity was observed between Guwahati and Nagpur, where the prevalence of moderate/high substance use risk differed by 35.1 percentage points (42.3% vs. 7.1%). These two sites were therefore selected for the subsequent decomposition analysis. Fairlie decomposition analysis showed that observed characteristics explained 37.9% of this disparity (Supplementary Table S17).

Table 3 presents the results of the multilevel ordinal logistic regression model examining factors associated with substance use risk severity. After adjustment for socio-demographic, socioeconomic, school-related, behavioural, health, and psychosocial characteristics, perceived accessibility of substances emerged as the strongest determinant of higher substance use risk. Compared with adolescents reporting low accessibility, those reporting moderate (aOR = 2.08, 95% CI: 1.72–2.51) and high accessibility (aOR = 1.75, 95% CI: 1.36–2.26) had greater odds of belonging to a higher risk category (Table 3).

**Table 3:** Results of the three-level multilevel ordinal logistic regression model showing adjusted odds ratios (aORs) and 95% confidence intervals for factors associated with substance use risk among adolescents (N = 5180).

Variable	Adjusted OR (95% CI)	p-value
Age group		
10–13 years	Ref	
14–16 years	0.86 (0.68–1.07)	0.18
17–19 years	0.76 (0.55–1.05)	0.09
Gender		
Female	Ref	
Male	0.74 (0.62–0.88)	0.001
Monthly family income		
Low income	Ref	
Middle income	1.11 (0.92–1.33)	0.26
High income	1.07 (0.87–1.31)	0.54
Household size		
≤5 members	Ref	
>5 members	1.03 (0.87–1.21)	0.75
Father's education		
Low	Ref	
Secondary	0.93 (0.77–1.12)	0.44
Higher	0.96 (0.68–1.36)	0.83
Mother's education		
Low	Ref	
Secondary	1.13 (0.94–1.37)	0.20
Higher	1.03 (0.68–1.57)	0.89
Religion		
Hindu	Ref	
Muslim	0.86 (0.68–1.10)	0.23
Others	0.90 (0.43–1.88)	0.78
Area of school		
Rural	Ref	
Urban	1.07 (0.69–1.67)	0.77
School type		
Government	Ref	
Private	0.94 (0.50–1.75)	0.84
Attitude toward substance use		
Low	Ref	
Moderate	0.96 (0.79–1.17)	0.69
High	1.30 (1.01–1.69)	0.046
Accessibility to substances		
Low	Ref	
Moderate	2.08 (1.72–2.51)	<0.001
High	1.75 (1.36–2.26)	<0.001
Digital exposure		
Low	Ref	
Moderate	1.29 (1.05–1.57)	0.013
High	1.44 (1.16–1.79)	0.001
Social engagement		
Low	Ref	
Moderate	0.92 (0.76–1.13)	0.45
High	0.98 (0.80–1.20)	0.85
Risky behavioural environment		
Low	Ref	
Moderate	1.05 (0.86–1.29)	0.61
High	1.03 (0.83–1.27)	0.82
Systolic blood pressure	1.00 (1.00–1.01)	0.07
Heart rate	1.00 (1.00–1.01)	0.65
Disability	1.09 (0.82–1.43)	0.56
Personality traits (SURPS domains)		
Hopelessness (z-score)	0.87 (0.76–0.99)	0.034
Anxiety sensitivity (z-score)	0.98 (0.88–1.09)	0.69
Impulsivity (z-score)	1.01 (0.90–1.13)	0.91
Sensation seeking (z-score)	1.02 (0.91–1.14)	0.78
Random-effects parameters		
Level	Variance (95% CI)	
Site	5.85 (1.70–20.14)	
School	1.31 (0.91–1.89)	

Odds ratios (OR) are presented with 95% confidence intervals (CI). aOR = adjusted odds ratio. Reference categories are indicated by (). Variables without aOR were not retained in the adjusted model. SBP = systolic blood pressure; bpm = beats per minute. SURPS domains are standardised (z-scores).

Higher digital exposure was also independently associated with elevated risk (moderate exposure: aOR = 1.29, 95% CI: 1.05–1.57; high exposure: aOR = 1.44, 95% CI: 1.16–1.79), as was a more favourable attitude towards substance use (aOR = 1.30, 95% CI: 1.01–1.69). Male adolescents had lower odds of higher substance use risk than females (aOR = 0.74, 95% CI: 0.62–0.88).

The multilevel model demonstrated improved fit compared with a null model (LR χ^2^ = 1312.77, p < 0.001), with statistically significant residual clustering at both school and site levels (Supplementary Table S5). Sensitivity analyses using categorical rather than continuous SURPS domains yielded similar findings, indicating the robustness of the primary results (Supplementary Table S6). Findings from substance-specific multilevel models were broadly consistent, with digital exposure emerging as the most consistent predictors across substance categories (Supplementary Tables S7–S15). Additionally, a sensitivity analysis excluding accessibility of substances from the model and including the full study sample (N = 6168) yielded broadly similar findings (Supplementary Table S19).

Among the examined domains, school characteristics accounted for the largest share of the explained difference (31.4%), followed by socio-demographic and economic factors (26.6%) and personality traits (19.5%) (Table 4). Attitudinal factors (7.6%), digital exposure (8.0%), and health-related factors (5.4%) made relatively smaller contributions, whereas social engagement and behavioural factors each contributed less than 1%.

**Table 4:** Domain-wise Fairlie decomposition of inter-site difference in moderate/high risk of substance use between Guwahati and Nagpur.

Variable	Coefficient (95% CI)	p-value	Total explained	% Contribution
Socio-demographic & economic factors			0.0355	26.60
Age	0.016 (−0.013, 0.045)	0.28		
Income	−0.006 (−0.014, 0.002)	0.16		
Members	0.002 (−0.002, 0.007)	0.33		
Father's education	−0.005 (−0.011, 0.001)	0.07		
Mother's education	0.007 (0.001, 0.015)	0.023		
Gender	−0.002 (−0.006, 0.001)	0.14		
Religion	0.023 (0.004, 0.045)	0.022		
School characteristics			0.0419	31.40
Area of school	0.001 (0.001, 0.002)	0.001		
Type of school	0.041 (−0.011, 0.097)	0.12		
Attitudinal factor			0.0102	7.62
Attitude score	0.010 (0.005, 0.015)	<0.001		
Accessibility of substances			0.0007	0.50
Accessibility score	−0.001 (−0.009, 0.007)	0.87		
Digital exposure			0.0107	7.99
Digital exposure mean	0.011 (−0.010, 0.031)	0.31		
Social engagement			0.0005	0.40
Social engagement scores	0.001 (0.001, 0.002)	<0.001		
Behavioural factors			0.0008	0.63
Risky behaviour towards substance use	0.001 (−0.021, 0.023)	0.94		
Health factors			0.0071	5.36
Systolic BP	−0.005 (−0.008, −0.001)	0.015		
Heart rate	0.006 (−0.006, 0.017)	0.33		
Disability	0.006 (0.003, 0.010)	<0.001		
Personality traits			0.0260	19.50
Hopelessness (HOP)	0.004 (−0.007, 0.015)	0.51		
Anxiety sensitivity (AS)	0.010 (0.001, 0.020)	0.049		
Impulsivity (IMP)	0.001 (−0.017, 0.016)	0.96		
Sensation seeking (SS)	0.011 (−0.003, 0.025)	0.10		
			0.1334	100

Positive coefficients indicate factors that widen the Guwahati–Nagpur disparity in moderate/high substance use risk, whereas negative coefficients indicate factors that reduce the disparity. Percentage contribution was calculated as total explained/0.133 × 100, where 0.133 represents the total explained difference. Percentages are based on absolute contributions and may not reflect the direction of effect.

Within the socio-demographic and economic domain, religion (β = 0.023; 95% CI: 0.004–0.045; p = 0.022) and maternal education (β = 0.007; 95% CI: 0.001–0.015; p = 0.023) were statistically significant contributors to the explained component of the disparity. Within the school characteristics domain, area of school was a statistically significant contributor (β = 0.001; 95% CI: 0.001–0.002; p = 0.001).

Among attitudinal and psychosocial factors, attitude towards substance use contributed significantly to the explained disparity (β = 0.010; 95% CI: 0.005–0.015; p < 0.001), while among personality traits, anxiety sensitivity was the only statistically significant contributor (β = 0.010; 95% CI: 0.001–0.020; p = 0.049).

## Discussion

This multi-centre study demonstrates substantial variation in adolescent substance use risk across six study sites in India. Using a standardised risk assessment framework, we found that substance use risk varied markedly across settings, with the highest prevalence observed in selected schools belonging to Guwahati and Deoghar and the lowest in Nagpur and Puducherry. Although these findings should be interpreted with caution considering the self-reported nature, they are consistent with previous studies from India that have documented variation in adolescent substance use, although most earlier studies focused on specific substances or single geographical areas.^16^^,^^18^^,^^23^ By examining multiple substances simultaneously across diverse settings, the present study extends existing evidence and highlights the uneven distribution of substance use risk among Indian adolescents. However, because the study was conducted in selected schools within six study sites and was not designed to generate state-level estimates, these findings should be interpreted as reflecting differences between the study sites rather than representative differences between the respective states.

The descriptive analyses revealed significant differences across study sites in accessibility of substances, digital exposure, attitudes towards substance use, and several socio-demographic characteristics. For example, adolescents in Guwahati reported the highest mean accessibility and digital exposure scores, whereas lower levels were generally observed in Nagpur. However, decomposition analysis demonstrated that accessibility contributed only minimally to the Guwahati–Nagpur disparity in substance use risk. This finding suggests that factors associated with individual-level substance use risk are not necessarily the same factors that explain geographical inequalities in risk. Therefore, while site-level differences were evident, the present study cannot determine the specific contextual mechanisms underlying these disparities.

The multilevel analysis identified accessibility of substances, digital exposure, and favourable attitudes towards substance use as important correlates of higher substance use risk. Adolescents reporting greater accessibility to substances had significantly higher odds of belonging to a higher risk category. Similarly, higher digital exposure was associated with elevated substance use risk, consistent with growing evidence that digital environments may influence substance-related perceptions and behaviours among adolescents. These findings indicate that substance use risk is associated with both physical and digital environmental exposures.^35^^,^^36^

Among the SURPS personality domains, hopelessness was the only trait significantly associated with substance use risk after adjustment, and the observed association was inverse. This finding contrasts with a substantial body of literature identifying externalising traits, particularly impulsivity and sensation seeking, as important predictors of substance use initiation, progression, and severity.^15^^,^^37^^,^^38^ Previous studies have consistently reported that adolescents with higher levels of impulsivity and sensation seeking are more likely to engage in experimentation and risk-taking behaviours.^15^^,^^38^^,^^39^ The absence of significant associations for impulsivity, sensation seeking, and anxiety sensitivity in the present study may reflect differences in population characteristics, self-reported data, the school-based nature of the sample, or attenuation of these effects after adjustment for behavioural and environmental factors. Given the unexpected direction of association for hopelessness and the lack of significant associations for other personality domains, these findings should be interpreted cautiously and warrant further investigation in longitudinal studies.

The Fairlie decomposition analysis provided additional insight into the observed inter-site disparities. Of the 35.1 percentage-point difference in moderate/high substance use risk between Guwahati and Nagpur, observed characteristics explained 37.9% of the gap. School characteristics accounted for the largest share of the explained component, followed by socio-demographic and economic factors and personality traits. Within the socio-demographic domain, religion and maternal education emerged as statistically significant contributors. However, these findings should not be interpreted as evidence of direct causal effects. Rather, they indicate that differences in these characteristics contributed to the observed disparity, while the underlying mechanisms remain unclear and may reflect broader social, cultural, familial, or contextual influences not captured in the study.

Importantly, nearly two-thirds of the Guwahati–Nagpur disparity remained unexplained by the variables included in the analysis. This finding suggests that additional factors not captured in the baseline survey may contribute to geographical differences in adolescent substance use risk. Potential explanations may include neighbourhood environments, peer substance use, school climate, local implementation of substance-control policies, and other contextual characteristics; however, these factors were not measured in the present study.^14^ Because these factors were not measured directly, their role remains speculative and should be explored in future studies. Consequently, the present findings should be interpreted as identifying associations rather than definitive explanations for inter-site inequalities in substance use risk.

The overall prevalence of moderate/high substance use risk observed in this study is broadly comparable with findings from previous school-based studies in India and low- and middle-income countries, although direct comparisons are complicated by differences in populations, settings, and measurement tools.^10^^,^^40^ Unlike many earlier studies that focused on single substances, the present analysis captured risk across multiple substance categories using a standardised assessment framework.^41^, ^42^, ^43^ Additionally, household income was not independently associated with substance use risk after adjustment. This finding may indicate that any association between socioeconomic circumstances and substance use risk is likely to be mediated through more proximal environmental, behavioural, or psychosocial factors.

The findings have important implications for adolescent substance use prevention in India. Accessibility of substances and digital exposure emerged as consistent correlates of higher substance use risk, suggesting that prevention strategies should extend beyond awareness-based approaches and consider environmental and digital risk factors. School-based programmes may benefit from incorporating substance use awareness, digital literacy, and screening approaches for identifying adolescents who may be at elevated risk.^40^

The substantial variation in substance use risk across study sites, together with the large unexplained component of the inter-site disparity, suggests that a uniform prevention approach may be insufficient. Instead, interventions should be adapted to local contexts and informed by region-specific risk profiles. Future prevention efforts should also consider broader influences, including family, peer, school, and community environments, which were not fully captured in the present study but may contribute to geographical inequalities in adolescent substance use risk.

This study has several limitations. First, the cross-sectional baseline design precludes establishing the temporal sequence between the measured characteristics and substance use risk. Consequently, the observed associations may be influenced by reverse causation or unmeasured confounding. Second, reliance on self-reported data may introduce social desirability bias, particularly for sensitive behaviours such as substance use. Third, recall bias may affect reporting of substance use patterns. Fourth, direct measures of neighbourhood characteristics and peer substance use were not available in the baseline survey dataset, which may partly explain the variation observed across study sites. Additionally, data on vaping/e-cigarette use and injectable drug use were not available in the dataset and could not be examined. Furthermore, responses to the accessibility-of-substances section were unavailable for the Puducherry site, resulting in missing accessibility data for participants from this study site. The inverse association observed between hopelessness and substance use risk was unexpected and contrasts with much of the existing literature; therefore, this finding should be interpreted cautiously and warrants further investigation in longitudinal and replication studies.

Furthermore, the very low number of adolescents classified as having moderate-to-high substance use risk in Puducherry resulted in insufficient outcome variation for stable Fairlie decomposition estimates. Consequently, Puducherry could not be included in the comparative decomposition analysis. Although a wide range of variables was included, a substantial proportion of inter-site variation remained unexplained, indicating the influence of unmeasured contextual factors and residual confounding. Moreover, while the Fairlie decomposition partitions observed differences into explained and unexplained components based on measured characteristics, the interpretive value of this approach is constrained by the completeness of the variables included; thus, the large unexplained component may reflect residual confounding rather than solely unobserved contextual influences. Accordingly, interpretations regarding site-specific socio-cultural norms, environmental influences, or differences in substance accessibility should be considered hypothesis-generating rather than confirmatory. Finally, although the multi-site design enhances diversity, the findings may not be generalisable to all adolescents in India, particularly out-of-school adolescents.

This secondary analysis of a multi-centre community-based intervention trial on substance use prevention highlights site-wise heterogeneity in distribution of self-reported substance use risk among school-going adolescents in India. The findings demonstrate that high-substance use risk is characterised by a convergence of adverse contextual conditions, including greater accessibility to substances, higher digital exposure, and risk-prone behavioural environments. Multivariable analysis identified environmental and behavioural factors as important correlates of substance use risk. Furthermore, decomposition analysis reveals that only a limited portion of inter-site disparities is explained by observed factors, underscoring the importance of unmeasured contextual and structural influences. These findings highlight the need for development of region-specific, school-based prevention strategies, including restricting local access to substances, integrating digital risk literacy into curricula, and strengthening peer-led interventions within the Ayushman Bharat School Health Programme. Targeting high-risk settings with combined environmental and behavioural approaches may reduce inequalities in adolescent substance use.

## Contributors

**UV**: Conceptualisation, Data verification, Funding acquisition, Methodology, Project administration, Resources, Supervision, Validation, Writing—review & editing. **MC**: Data curation, Formal analysis, Methodology, Visualisation, Writing—original draft, Writing—review & editing. **PC**: Project administration, Supervision, Validation, Writing—review & editing. **KJ**: Project administration, Supervision, Writing—review & editing. **SB**: Project administration, Validation, Writing—review & editing. **APG**: Project administration, Supervision, Validation, Writing—review & editing. **RA**: Project administration, Supervision, Validation, Writing—review & editing. **RT**: Validation, Writing—review & editing. **VR**: Validation, Writing—review & editing. **AMD**: Investigation, Project administration, Validation.

## Data sharing statement

All relevant de-identified data used in the study are available in the cross-disciplinary repository Kaggle, accessible through the link: https://doi.org/10.34740/kaggle/dsv/15927146.

## Editor note

The Lancet Group takes a neutral position with respect to territorial claims in published maps and institutional affiliations.

## Declaration of interests

The authors declare that they have no known competing financial interests or personal relationships that could have appeared to influence the work reported in this paper.
