# Supplementary material for: Substance use risk among school going adolescents in India: secondary analysis of Community Based Peer-Led Intervention (CPLI) trial
Source: Lancet Reg Health Southeast Asia. 2026 Jul 22;51:100821. doi: 10.1016/j.lansea.2026.100821 (PMC13396925; doi:10.1016/j.lansea.2026.100821)
Supplement: Supplementary Figure and Tables [file mmc1.docx]

**Supplementary Table 1: Independent variables and their construction**

| **Variables** | **Description** |
| --- | --- |
| **Socio-demographic and economic factors** | |
| Age | Continuous (years). For analytical purposes, participants were categorised into early adolescence (10–13 years), middle adolescence (14–16 years), and late adolescence (17–19 years) to account for developmental differences across the adolescent age spectrum. |
| Gender | Male / Female / Other |
| Religion | Hindu / Muslim / Others |
| Household size | Number of household members residing in the participant's household; recoded as ≤5 members and >5 members for analysis. |
| Monthly household income | Reported monthly household income was categorized into tertiles (low-, middle-, and high-income groups) based on the distribution of the study population and used as a proxy measure of household socioeconomic status. |
| Father’s education | Highest level of education attained by the father, recoded into three categories: primary or below, secondary education, and higher education. |
| Mother’s education | Highest level of education attained by the mother, recoded into three categories: primary or below, secondary education, and higher education. |
| **School characteristics** | |
| Area of school | Rural and urban |
| Type of school | Public (government) and private. |
| **Attitudinal factor** | |
| Attitude towards substance use | Attitude towards substance use was measured using a set of 12 items capturing adolescents’ beliefs and perceptions regarding the use of tobacco, alcohol, and other substances. The items assessed normative beliefs, perceived social acceptability, coping mechanisms, and cultural or community norms related to substance use. Respondents were asked to indicate their level of agreement with each statement on a five-point Likert scale ranging from strongly disagree (1) to strongly agree (5).  All items were framed in a manner such that higher scores reflected stronger anti-substance use attitudes. A composite attitude score was computed by taking the mean of the responses across the twelve items for each participant, ensuring equal weighting of all items.  The continuous mean score was subsequently categorised into three groups: low, moderate, and high anti-substance use attitude, using tertile distribution. |
| **Accessibility factor** | |
| Accessibility to substances | Accessibility to substances was measured using five items assessing the perceived ease of obtaining commonly used substances and exposure to substance use in the immediate neighbourhood. The items included perceived ease of access to cigarettes/bidi, alcohol, gutka/tobacco products, and cannabis (ganja/marijuana), along with the frequency of observing substance use (e.g., smoking or drinking) in the surrounding environment.  Responses to each item were recorded on a five-point Likert scale ranging from very difficult (1) to very easy (5), with higher scores indicating greater accessibility and environmental exposure to substances. A composite accessibility score was generated by calculating the mean of the responses across the five items for each participant.  The continuous mean score was subsequently categorised into three groups: low, medium, and high accessibility, based on the interquartile range distribution. Participants with scores in the lower quartile were classified as having low accessibility, those within the interquartile range as having medium accessibility, and those in the upper quartile as having high accessibility. |
| **Digital exposure** | |
| Digital exposure | Digital exposure was assessed using four items capturing different dimensions of adolescents’ engagement with digital media, including time spent on social media platforms, exposure to substance-related content, late-night use of digital devices for entertainment, and perceived pressure from social media to try new experiences or conform to trends. Each item was measured on a five-point Likert scale, with higher values indicating greater exposure or influence.  Responses were coded such that higher scores consistently reflected greater digital exposure and potential risk. A composite digital exposure score was generated by calculating the mean of the responses across the four items for each participant, ensuring equal contribution of each dimension.  The continuous mean score was subsequently categorised into three groups, low, moderate, and high digital exposure, using tertile distribution. |
| **Social engagement** | |
| Social engagement | Social engagement was measured to capture adolescents’ involvement in various social, recreational, and lifestyle-related activities. This variable was constructed using five items reflecting participation in school sports or physical activities, involvement in cultural activities such as dance, music, drama, or art, engagement in meaningful conversations with family members, participation in hobbies or creative pursuits (such as reading, writing, or crafts), and involvement in community or youth group activities.  Each item was assessed using a five-point Likert scale ranging from never (1) to always (5). Higher scores indicate greater participation in these activities, reflecting better social engagement and healthier lifestyle practices, while lower scores indicate limited participation and poorer engagement.  A composite social engagement score was generated by calculating the mean of responses across the five items for each participant. The resulting continuous score was then categorised into three groups: low, moderate, and high social engagement, using tertile distribution. |
| **Behavioural factors** | |
| Risky behaviour towards substance use index | Risky behaviour towards substance use was assessed using a composite index constructed from twelve items capturing adolescents’ behavioural patterns, exposure to substance-use environments, supervision, communication, coping mechanisms, peer influence, and access to financial resources. These items included behaviours such as spending time in environments where substance use occurs, staying out late without parental knowledge, skipping school, exposure to substance use in the neighbourhood, as well as behaviours such as seeking support during stress, avoiding substance-related media, and parental supervision.  Each item was measured using ordinal response categories and subsequently recoded such that higher values consistently reflected higher risk behaviour. This ensured uniform directionality across all items, where behaviours indicative of greater exposure, lower supervision, poorer coping, and increased opportunity for substance use were assigned higher scores.  A composite behavioural risk score was then generated by summing the recoded values across all twelve items for each participant. Higher total scores indicate a greater overall propensity towards risk-related behaviours associated with substance use.  The continuous composite score was further categorised into three groups: low, moderate, and high-risk behaviour, using tertile distribution. This classification reflects relative differences in behavioural risk profiles among adolescents within the study population and was used for subsequent analysis. |
| **Health conditions** | |
| Systolic blood pressure (SBP) | Continuous (mmHg). Measured using a digital blood pressure monitor (Dr. Morepen BP-14); two readings were taken after rest, and the second systolic blood pressure measurement (mmHg) was used for analysis. |
| Heart rate | Continuous (beats per minute). Measured concurrently using the digital blood pressure monitor (Dr. Morepen BP-14); heart rate (beats per minute) recorded during the second blood pressure assessment was used for analysis. |
| Disability status | Binary variable (0 = no disability, 1 = any disability among these Physical disability (e.g., difficulty walking, using hands, etc, vision impairment (even after wearing glasses), hearing impairment (even with hearing aid), speech difficulty, intellectual or learning disability, mental health condition (e.g., depression, anxiety, etc.), multiple disabilities, or other. |
| **Personality traits: Substance Use Risk Profile Scale (SURPS)** | |
| Hopelessness | The hopelessness domain was constructed using items from the SURPS that capture negative affect, pessimistic thinking, and feelings of low self-worth. Responses to all items corresponding to this domain were first reviewed for directionality, and negatively worded items were reverse-coded where necessary to ensure consistency. The final hopelessness score was then calculated by summing the responses across all relevant items, with higher scores indicating greater levels of hopelessness. In Table 1 values for hopelessness was represented in raw scores (mean ± SD). To facilitate comparability with other personality domains, the total score was standardized into a z-score with a mean of zero and a standard deviation of one, and these z-scores have been used in regression. |
| Anxiety sensitivity | The anxiety sensitivity domain reflects the extent to which individuals fear anxiety-related sensations and their potential consequences. Items corresponding to this domain were identified from the SURPS instrument, and appropriate reverse coding was applied to negatively phrased items. A composite score was generated by summing responses across all anxiety sensitivity items, with higher values representing greater sensitivity to anxiety symptoms. In Table 1 values for anxiety sensitivity was represented in raw scores (mean ± SD). The score was standardized into a z-score and included in regression models. |
| Impulsivity | Impulsivity was measured using SURPS items that assess tendencies toward rapid, unplanned reactions and difficulty in behavioral control. After ensuring correct coding direction through reverse coding of selected items, responses were aggregated by summing across all impulsivity-related items. Higher scores on this composite measure indicate greater impulsive tendencies. In Table 1 values for impulsivity was represented in raw scores (mean ± SD). The total score was standardized into a z-score and used in regression analyses. |
| Sensation seeking | The sensation seeking domain captures the tendency to pursue novel, exciting, and potentially risky experiences. Relevant items from the SURPS scale were used to construct this domain, with reverse coding applied where necessary to maintain consistency in scoring direction. A total sensation seeking score was calculated by summing responses across all items within this domain, with higher scores indicating stronger sensation-seeking traits. In Table 1 values for sensation seeking was represented in raw scores (mean ± SD). The total score was standardized into a z-score and used in regression analyses. |

**Supplementary Table 2: Internal consistency reliability of study scales**

| **Scale / Construct** | **Number of items** | **Average inter-item covariance** | **Cronbach’s alpha** |
| --- | --- | --- | --- |
| Anti-substance use attitude | 12 | 0.631 | 0.926 |
| Accessibility to substances | 5 | 1.279 | 0.914 |
| Digital exposure | 4 | 0.497 | 0.719 |
| Social engagement | 5 | 0.720 | 0.766 |
| Risky behaviour towards substance use | 12 | 0.198 | 0.618 |
| **SURPS domains** |  |  |  |
| Hopelessness | 6 | 1.097 | 0.888 |
| Anxiety Sensitivity | 5 | 0.559 | 0.660 |
| Impulsivity | 5 | 0.405 | 0.650 |
| Sensation Seeking | 6 | 0.373 | 0.767 |

**Supplementary Table 3. Bonferroni-adjusted pairwise comparisons of moderate/high substance use risk across study sites**

| **Comparison** | **Contrast** | **SE** | **Unadjusted p-value** | **Bonferroni-adjusted p-value** | **Bonferroni-adjusted 95% CI** |
| --- | --- | --- | --- | --- | --- |
| Guwahati vs Gorakhpur | 0.811 | 0.095 | <0.001 | <0.001 | 0.532 to 1.090 |
| Nagpur vs Gorakhpur | -1.445 | 0.136 | <0.001 | <0.001 | -1.843 to -1.047 |
| Rajkot vs Gorakhpur | -0.734 | 0.117 | <0.001 | <0.001 | -1.078 to -0.390 |
| Deoghar vs Gorakhpur | 0.771 | 0.091 | <0.001 | <0.001 | 0.504 to 1.038 |
| Puducherry vs Gorakhpur | -5.771 | 1.003 | <0.001 | <0.001 | -8.714 to -2.828 |
| Nagpur vs Guwahati | -2.256 | 0.136 | <0.001 | <0.001 | -2.656 to -1.856 |
| Rajkot vs Guwahati | -1.545 | 0.118 | <0.001 | <0.001 | -1.892 to -1.199 |
| Deoghar vs Guwahati | -0.040 | 0.092 | 0.66 | 1.00 | -0.310 to 0.230 |
| Rajkot vs Nagpur | 0.711 | 0.153 | <0.001 | <0.001 | 0.263 to 1.159 |
| Deoghar vs Nagpur | 2.216 | 0.134 | <0.001 | <0.001 | 1.824 to 2.609 |
| Puducherry vs Nagpur | -4.326 | 1.007 | <0.001 | <0.001 | -7.283 to -1.369 |
| Deoghar vs Rajkot | 1.505 | 0.115 | <0.001 | <0.001 | 1.168 to 1.843 |
| Puducherry vs Rajkot | -5.037 | 1.005 | <0.001 | <0.001 | -7.987 to -2.086 |
| Puducherry vs Deoghar | -6.542 | 1.002 | <0.001 | <0.001 | -9.484 to -3.600 |

**Note:** Pairwise comparisons were performed following the overall likelihood ratio χ² test using Bonferroni correction to account for multiple comparisons. Positive contrast values indicate a higher log-odds of moderate/high substance use risk in the first-listed site relative to the second-listed site, whereas negative values indicate lower log-odds. Both unadjusted and Bonferroni-adjusted p-values are presented. Bonferroni-adjusted 95% confidence intervals are reported. After Bonferroni correction, only the comparison between Guwahati and Deoghar was not statistically significant (adjusted p = 1.00), whereas all other pairwise comparisons remained statistically significant. Given the extremely low prevalence of moderate/high substance use risk in Puducherry (0.1%), comparisons involving Puducherry should be interpreted cautiously.

**Supplementary Table 4: Variance inflation factors**

| **Variable** | **VIF** | **1/VIF** |
| --- | --- | --- |
| **Gender** |  |  |
| Female | Ref |  |
| Male | 1.13 | 0.89 |
| **Monthly family income** |  |  |
| Low income | Ref |  |
| Middle income | 1.27 | 0.79 |
| High income | 1.41 | 0.71 |
| **Household size** |  |  |
| ≤5 members | Ref |  |
| >5 members | 1.06 | 0.95 |
| **Father's education** |  |  |
| Low | Ref |  |
| Secondary | 1.32 | 0.76 |
| Higher | 1.45 | 0.69 |
| **Mother's education** |  |  |
| Low | Ref |  |
| Secondary | 1.32 | 0.76 |
| Higher | 1.44 | 0.69 |
| **Religion** |  |  |
| Hindu | Ref |  |
| Muslim | 1.06 | 0.94 |
| Others | 1.03 | 0.98 |
| **Area of school** |  |  |
| Rural | Ref |  |
| Urban | 1.05 | 0.95 |
| **School type** |  |  |
| Government | Ref |  |
| Private | 1.27 | 0.79 |
| **Attitude toward substance use** |  |  |
| Low | Ref |  |
| Moderate | 1.51 | 0.66 |
| High | 1.67 | 0.60 |
| **Accessibility to substances** |  |  |
| Low | Ref |  |
| Moderate | 1.47 | 0.68 |
| High | 1.95 | 0.51 |
| **Digital exposure** |  |  |
| Low | Ref |  |
| Moderate | 1.34 | 0.75 |
| High | 1.54 | 0.65 |
| **Social engagement** |  |  |
| Low | Ref |  |
| Moderate | 1.28 | 0.78 |
| High | 1.36 | 0.74 |
| **Risky behavioural environment** |  |  |
| Low | Ref |  |
| Moderate | 1.42 | 0.70 |
| High | 1.52 | 0.66 |
| **Systolic blood pressure** | 1.77 | 0.56 |
| **Heart rate** | 1.73 | 0.58 |
| **Disability** |  |  |
| No | Ref |  |
| Yes | 1.02 | 0.98 |
| Hopelessness (z-score) | 1.39 | 0.72 |
| Anxiety sensitivity (z-score) | 1.46 | 0.69 |
| Impulsivity (z-score) | 1.80 | 0.56 |
| Sensation seeking (z-score) | 1.68 | 0.60 |
| **Mean VIF** | 1.40 |  |

**Supplementary Table 5. Multilevel model building and variance components**

| **Parameter** | **Null Model (Model 0)** | **Adjusted Model (Model 1)** |
| --- | --- | --- |
| Log likelihood | -3222.80 | -2892.38 |
| AIC | 6451.59 | 5854.75 |
| BIC | 6472.00 | 6088.40 |
| LR test vs conventional ordinal logistic regression | χ²=1779.20, p<0.001 | χ²=1312.77, p<0.001 |
| Site-level variance | 5.34 (1.53–18.65) | 5.85 (1.70–20.14) |
| School-level variance | 1.32 (0.94–1.87) | 1.31 (0.91–1.89) |

**Supplementary Table 6. Sensitivity analysis comparing continuous versus categorical SURPS domains in the multilevel ordinal logistic regression model for combined substance use risk**

| **SURPS Domain** | **Primary Model (Continuous z-score)  aOR (95% CI)** | **p-value** | **Sensitivity Model (Tertiles)  aOR (95% CI)** | **p-value** |
| --- | --- | --- | --- | --- |
| **Hopelessness** | 0.87 (0.76–0.99) | 0.034 | Moderate vs Low: 0.99 (0.81–1.20) | 0.88 |
|  |  |  | High vs Low: 0.94 (0.74–1.19) | 0.61 |
| **Anxiety Sensitivity** | 0.98 (0.88–1.09) | 0.69 | Moderate vs Low: 0.95 (0.78–1.15) | 0.61 |
|  |  |  | High vs Low: 1.14 (0.93–1.46) | 0.19 |
| **Impulsivity** | 1.01 (0.90–1.13) | 0.91 | Moderate vs Low: 0.89 (0.72–1.08) | 0.23 |
|  |  |  | High vs Low: 0.86 (0.68–1.09) | 0.22 |
| **Sensation Seeking** | 1.02 (0.91–1.14) | 0.78 | Moderate vs Low: 1.06 (0.87–1.28) | 0.57 |
|  |  |  | High vs Low: 1.12 (0.88–1.42) | 0.37 |

Note: Adjusted odds ratios (aORs) were estimated using three-level multilevel ordinal logistic regression models with random intercepts for study site and school. All models were adjusted for age group, gender, household income tertile, household size, father's education, mother's education, religion, school area, school type, attitude toward substance use, accessibility, digital exposure, social engagement, behavioural risk environment, systolic blood pressure, heart rate, disability status, and the four SURPS personality domains (hopelessness, anxiety sensitivity, impulsivity, and sensation seeking). In the primary model, SURPS domains were included as standardized continuous z-scores, whereas in the sensitivity analysis they were categorized into tertiles (low, moderate, and high).

**Supplementary Table 7. Multilevel ordinal logistic regression analysis of tobacco use risk among adolescents (N=5,180)**

| **Variable** | **aOR** | **95% CI** | **p-value** |
| --- | --- | --- | --- |
| **Age group** |  |  |  |
| 10–13 years | Ref | – | – |
| 14–16 years | 0.79 | 0.61–1.02 | 0.07 |
| 17–19 years | 0.63 | 0.43–0.92 | 0.018 |
| **Gender** |  |  |  |
| Female | Ref | – | – |
| Male | 0.75 | 0.61–0.92 | 0.006 |
| **Household income tertile** |  |  |  |
| Low income | Ref | – | – |
| Middle income | 1.12 | 0.90–1.40 | 0.31 |
| High income | 1.09 | 0.85–1.40 | 0.49 |
| **Household size** |  |  |  |
| ≤5 members | Ref | – | – |
| >5 members | 1.05 | 0.86–1.30 | 0.62 |
| **Father's education** |  |  |  |
| Primary or below | Ref | – | – |
| Secondary | 1.07 | 0.86–1.33 | 0.52 |
| Higher education | 1.23 | 0.82–1.85 | 0.31 |
| **Mother's education** |  |  |  |
| Primary or below | Ref | – | – |
| Secondary | 1.01 | 0.81–1.25 | 0.98 |
| Higher education | 1.06 | 0.65–1.74 | 0.82 |
| **Religion** |  |  |  |
| Hindu | Ref | – | – |
| Muslim | 1.02 | 0.78–1.33 | 0.89 |
| Others | 0.88 | 0.36–2.17 | 0.78 |
| **School area** |  |  |  |
| Rural | Ref | – | – |
| Urban | 1.19 | 0.71–1.99 | 0.51 |
| **School type** |  |  |  |
| Government | Ref | – | – |
| Private | 0.83 | 0.42–1.66 | 0.60 |
| **Attitude toward substance use** |  |  |  |
| Low | Ref | – | – |
| Moderate | 0.89 | 0.70–1.13 | 0.35 |
| High | 1.33 | 0.97–1.83 | 0.07 |
| **Accessibility score** |  |  |  |
| Low | Ref | – | – |
| Moderate | 2.28 | 1.78–2.92 | <0.001 |
| High | 2.24 | 1.64–3.06 | <0.001 |
| **Digital exposure** |  |  |  |
| Low | Ref | – | – |
| Moderate | 1.47 | 1.14–1.89 | 0.003 |
| High | 1.45 | 1.10–1.92 | 0.009 |
| **Social engagement** |  |  |  |
| Low | Ref | – | – |
| Moderate engagement | 1.06 | 0.83–1.35 | 0.65 |
| High engagement | 1.12 | 0.87–1.44 | 0.38 |
| **Behavioural risk environment** |  |  |  |
| Low risk | Ref | – | – |
| Moderate risk | 0.94 | 0.74–1.20 | 0.61 |
| High risk | 0.82 | 0.63–1.06 | 0.13 |
| **Systolic blood pressure (continuous)** | 1.00 | 1.00–1.01 | 0.16 |
| **Heart rate (continuous)** | 1.00 | 0.99–1.01 | 0.94 |
| **Disability status** |  |  |  |
| No disability | Ref | – | – |
| Has disability | 0.81 | 0.56–1.16 | 0.25 |
| Hopelessness (z-score) | 0.81 | 0.69–0.94 | 0.008 |
| Anxiety sensitivity (z-score) | 0.98 | 0.86–1.11 | 0.75 |
| Impulsivity (z-score) | 1.02 | 0.88–1.18 | 0.81 |
| Sensation seeking (z-score) | 0.98 | 0.86–1.13 | 0.80 |
| **Random-effects estimates** |  |  |  |
| **Level** | **Variance (95% CI)** | | |
| Site | 5.30 (1.57–17.89) | | |
| School | 1.62 (1.09–2.41) | | |

**Supplementary Table 8: Multilevel ordinal logistic regression analysis of alcohol use risk among adolescents (N=5,180)**

| **Variable** | **aOR** | **95% CI** | **p-value** |
| --- | --- | --- | --- |
| **Age group** | |  |  |
| 10–13 years | Ref | – | – |
| 14–16 years | 0.69 | 0.51–0.94 | 0.02 |
| 17–19 years | 0.52 | 0.33–0.83 | 0.006 |
| **Gender** |  |  |  |
| Female | Ref | – | – |
| Male | 0.62 | 0.48–0.80 | <0.001 |
| **Household income tertile** | | |  |
| Low income | Ref | – | – |
| Middle income | 1.15 | 0.88–1.50 | 0.32 |
| High income | 1.48 | 1.08–2.02 | 0.014 |
| **Household size** | |  |  |
| ≤5 members | Ref | – | – |
| >5 members | 1.13 | 0.89–1.45 | 0.32 |
| **Father's education** | |  |  |
| Primary or below | Ref | – | – |
| Secondary | 1.01 | 0.78–1.31 | 0.94 |
| Higher education | 1.29 | 0.80–2.08 | 0.30 |
| **Mother's education** | |  |  |
| Primary or below | Ref | – | – |
| Secondary | 1.07 | 0.82–1.40 | 0.60 |
| Higher education | 1.15 | 0.64–2.07 | 0.65 |
| **Religion** |  |  |  |
| Hindu | Ref | – | – |
| Muslim | 0.84 | 0.60–1.18 | 0.32 |
| Others | 1.28 | 0.45–3.65 | 0.65 |
| **School area** | |  |  |
| Rural | Ref | – | – |
| Urban | 1.14 | 0.58–2.26 | 0.70 |
| **School type** | |  |  |
| Government | Ref | – | – |
| Private | 0.49 | 0.21–1.12 | 0.09 |
| **Attitude toward substance use** | | | |
| Low | Ref | – | – |
| Moderate | 1.24 | 0.93–1.65 | 0.15 |
| High | 2.15 | 1.47–3.15 | <0.001 |
| **Accessibility score** |  |  |  |
| Low | Ref | – | – |
| Moderate | 2.57 | 1.92–3.45 | <0.001 |
| High | 3.07 | 2.12–4.44 | <0.001 |
| **Digital exposure** | |  |  |
| Low | Ref | – | – |
| Moderate | 1.46 | 1.09–1.97 | 0.012 |
| High | 1.67 | 1.20–2.33 | 0.003 |
| **Social engagement** | |  |  |
| Low | Ref | – | – |
| Moderate engagement | 1.13 | 0.84–1.52 | 0.41 |
| High engagement | 1.11 | 0.82–1.51 | 0.50 |
| **Behavioural risk environment** | | |  |
| Low risk | Ref | – | – |
| Moderate risk | 0.80 | 0.60–1.07 | 0.14 |
| High risk | 0.69 | 0.50–0.94 | 0.020 |
| **Systolic blood pressure (continuous)** | 1.00 | 1.00–1.01 | 0.26 |
| **Heart rate (continuous)** | 1.00 | 0.99–1.01 | 0.64 |
| **Disability status** | |  |  |
| No disability | Ref | – | – |
| Has disability | 0.82 | 0.55–1.23 | 0.34 |
| Hopelessness (z-score) | 0.82 | 0.68–0.99 | 0.035 |
| Anxiety sensitivity (z-score) | 1.04 | 0.89–1.21 | 0.64 |
| Impulsivity (z-score) | 0.96 | 0.81–1.14 | 0.63 |
| Sensation seeking (z-score) | 0.98 | 0.83–1.15 | 0.81 |
| **Random-effects estimates** | | | |
| **Level** | **Variance (95% CI)** | | |
| Site | 11.12 (3.11–39.79) | | |
| School | 2.50 (1.62–3.87) | | |

**Supplementary Table 9. Multilevel ordinal logistic regression analysis of cannabis use risk among adolescents (N=5,180)**

| **Variable** | **aOR** | **95% CI** | **p-value** |
| --- | --- | --- | --- |
| **Age group** | |  |  |
| 10–13 years | Ref | – | – |
| 14–16 years | 0.78 | 0.59–1.04 | 0.09 |
| 17–19 years | 0.61 | 0.40–0.93 | 0.02 |
| **Gender** |  |  |  |
| Female | Ref | – | – |
| Male | 0.63 | 0.51–0.80 | <0.001 |
| **Household income tertile** | | |  |
| Low income | Ref | – | – |
| Middle income | 1.09 | 0.86–1.39 | 0.46 |
| High income | 1.23 | 0.93–1.63 | 0.14 |
| **Household size** | |  |  |
| ≤5 members | Ref | – | – |
| >5 members | 1.12 | 0.90–1.39 | 0.32 |
| **Father's education** | |  |  |
| Primary or below | Ref | – | – |
| Secondary | 0.95 | 0.75–1.20 | 0.68 |
| Higher education | 0.99 | 0.64–1.55 | 0.97 |
| **Mother's education** | |  |  |
| Primary or below | Ref | – | – |
| Secondary | 1.12 | 0.88–1.43 | 0.35 |
| Higher education | 1.29 | 0.76–2.19 | 0.35 |
| **Religion** |  |  |  |
| Hindu | Ref | – | – |
| Muslim | 0.86 | 0.63–1.18 | 0.36 |
| Others | 0.66 | 0.23–1.90 | 0.44 |
| **School area** | |  |  |
| Rural | Ref | – | – |
| Urban | 1.22 | 0.68–2.19 | 0.50 |
| **School type** | |  |  |
| Government | Ref | – | – |
| Private | 0.63 | 0.29–1.35 | 0.23 |
| **Attitude toward substance use** | | | |
| Low | Ref | – | – |
| Moderate | 0.98 | 0.76–1.27 | 0.89 |
| High | 1.58 | 1.13–2.22 | 0.008 |
| **Accessibility score** |  |  |  |
| Low | Ref | – | – |
| Moderate | 2.53 | 1.96–3.26 | <0.001 |
| High | 2.42 | 1.74–3.36 | <0.001 |
| **Digital exposure** | |  |  |
| Low | Ref | – | – |
| Moderate | 1.25 | 0.96–1.62 | 0.09 |
| High | 1.38 | 1.03–1.84 | 0.029 |
| **Social engagement** | |  |  |
| Low | Ref | – | – |
| Moderate engagement | 0.94 | 0.72–1.22 | 0.63 |
| High engagement | 1.01 | 0.77–1.32 | 0.96 |
| **Behavioural risk environment** | | |  |
| Low risk | Ref | – | – |
| Moderate risk | 0.95 | 0.73–1.23 | 0.70 |
| High risk | 0.79 | 0.60–1.04 | 0.09 |
| **Systolic blood pressure (continuous)** | 1.01 | 1.00–1.01 | 0.13 |
| **Heart rate (continuous)** | 1.00 | 0.99–1.01 | 0.71 |
| **Disability status** | |  |  |
| No disability | Ref | – | – |
| Has disability | 0.89 | 0.63–1.27 | 0.53 |
| Hopelessness (z-score) | 0.85 | 0.72–1.01 | 0.06 |
| Anxiety sensitivity (z-score) | 1.02 | 0.89–1.17 | 0.75 |
| Impulsivity (z-score) | 0.96 | 0.82–1.12 | 0.61 |
| Sensation seeking (z-score) | 0.98 | 0.85–1.13 | 0.76 |
| **Random-effects estimates** | | | |
| **Level** | **Variance (95% CI)** | | |
| Site | 9.02 (2.41–33.81) | | |
| School | 1.88 (1.24–2.84) | | |

**Supplementary Table 10. Multilevel ordinal logistic regression analysis of cocaine use risk among adolescents (N=5,180)**

| **Variable** | **aOR** | **95% CI** | **p-value** |
| --- | --- | --- | --- |
| **Age group** | |  |  |
| 10–13 years | Ref | – | – |
| 14–16 years | 0.73 | 0.56–0.97 | 0.029 |
| 17–19 years | 0.60 | 0.40–0.91 | 0.015 |
| **Gender** |  |  |  |
| Female | Ref | – | – |
| Male | 0.68 | 0.54–0.85 | 0.001 |
| **Household income tertile** | | |  |
| Low income | Ref | – | – |
| Middle income | 1.10 | 0.86–1.39 | 0.45 |
| High income | 1.15 | 0.87–1.51 | 0.34 |
| **Household size** | |  |  |
| ≤5 members | Ref | – | – |
| >5 members | 1.05 | 0.84–1.30 | 0.67 |
| **Father's education** | |  |  |
| Primary or below | Ref | – | – |
| Secondary | 1.04 | 0.83–1.32 | 0.72 |
| Higher education | 1.11 | 0.72–1.73 | 0.64 |
| **Mother's education** | |  |  |
| Primary or below | Ref | – | – |
| Secondary | 1.14 | 0.89–1.44 | 0.30 |
| Higher education | 1.22 | 0.73–2.06 | 0.45 |
| **Religion** |  |  |  |
| Hindu | Ref | – | – |
| Muslim | 0.88 | 0.64–1.21 | 0.43 |
| Others | 0.90 | 0.36–2.26 | 0.82 |
| **School area** | |  |  |
| Rural | Ref | – | – |
| Urban | 1.01 | 0.56–1.82 | 0.96 |
| **School type** | |  |  |
| Government | Ref | – | – |
| Private | 0.55 | 0.26–1.20 | 0.13 |
| **Attitude toward substance use** | | | |
| Low | Ref | – | – |
| Moderate | 0.96 | 0.74–1.23 | 0.73 |
| High | 1.50 | 1.07–2.09 | 0.017 |
| **Accessibility score** |  |  |  |
| Low | Ref | – | – |
| Moderate | 2.26 | 1.77–2.90 | <0.001 |
| High | 1.91 | 1.38–2.65 | <0.001 |
| **Digital exposure** | |  |  |
| Low | Ref | – | – |
| Moderate | 1.26 | 0.98–1.63 | 0.07 |
| High | 1.42 | 1.06–1.89 | 0.018 |
| **Social engagement** | |  |  |
| Low | Ref | – | – |
| Moderate engagement | 1.01 | 0.77–1.29 | 0.99 |
| High engagement | 1.08 | 0.83–1.41 | 0.57 |
| **Behavioural risk environment** | | |  |
| Low risk | Ref | – | – |
| Moderate risk | 0.98 | 0.76–1.26 | 0.87 |
| High risk | 0.89 | 0.67–1.17 | 0.40 |
| **Systolic blood pressure (continuous)** | 1.01 | 1.00–1.01 | 0.031 |
| **Heart rate (continuous)** | 1.00 | 0.99–1.01 | 0.99 |
| **Disability status** | |  |  |
| No disability | Ref | – | – |
| Has disability | 0.94 | 0.66–1.33 | 0.72 |
| Hopelessness (z-score) | 0.87 | 0.74–1.03 | 0.10 |
| Anxiety sensitivity (z-score) | 0.98 | 0.86–1.12 | 0.79 |
| Impulsivity (z-score) | 0.98 | 0.84–1.14 | 0.76 |
| Sensation seeking (z-score) | 0.98 | 0.85–1.13 | 0.80 |
| **Random-effects estimates** | | | |
| **Level** | **Variance (95% CI)** | | |
| Site | 8.51 (2.27–31.94) | | |
| School | 1.94 (1.29–2.94) | | |

**Supplementary Table 11. Multilevel ordinal logistic regression analysis of amphetamine use risk among adolescents (N=5,180)**

| **Variable** | **aOR** | **95% CI** | **p-value** |
| --- | --- | --- | --- |
| **Age group** | |  |  |
| 10–13 years | Ref | – | – |
| 14–16 years | 0.77 | 0.59–1.02 | 0.07 |
| 17–19 years | 0.55 | 0.37–0.84 | 0.005 |
| **Gender** |  |  |  |
| Female | Ref | – | – |
| Male | 0.66 | 0.53–0.83 | <0.001 |
| **Household income tertile** | | |  |
| Low income | Ref | – | – |
| Middle income | 1.05 | 0.83–1.33 | 0.68 |
| High income | 1.05 | 0.80–1.39 | 0.70 |
| **Household size** | |  |  |
| ≤5 members | Ref | – | – |
| >5 members | 1.06 | 0.86–1.31 | 0.58 |
| **Father's education** | |  |  |
| Primary or below | Ref | – | – |
| Secondary | 0.94 | 0.74–1.19 | 0.62 |
| Higher education | 1.01 | 0.66–1.57 | 0.95 |
| **Mother's education** | |  |  |
| Primary or below | Ref | – | – |
| Secondary | 1.11 | 0.88–1.42 | 0.38 |
| Higher education | 1.50 | 0.89–2.52 | 0.13 |
| **Religion** |  |  |  |
| Hindu | Ref | – | – |
| Muslim | 0.85 | 0.62–1.16 | 0.30 |
| Others | 0.70 | 0.24–2.03 | 0.51 |
| **School area** | |  |  |
| Rural | Ref | – | – |
| Urban | 1.15 | 0.64–2.04 | 0.64 |
| **School type** | |  |  |
| Government | Ref | – | – |
| Private | 0.50 | 0.23–1.10 | 0.09 |
| **Attitude toward substance use** | | | |
| Low | Ref | – | – |
| Moderate | 1.06 | 0.82–1.38 | 0.63 |
| High | 1.93 | 1.38–2.68 | <0.001 |
| **Accessibility score** |  |  |  |
| Low | Ref | – | – |
| Moderate | 2.46 | 1.92–3.15 | <0.001 |
| High | 2.01 | 1.46–2.78 | <0.001 |
| **Digital exposure** | |  |  |
| Low | Ref | – | – |
| Moderate | 1.38 | 1.07–1.78 | 0.012 |
| High | 1.44 | 1.09–1.92 | 0.011 |
| **Social engagement** | |  |  |
| Low | Ref | – | – |
| Moderate engagement | 1.02 | 0.79–1.31 | 0.87 |
| High engagement | 0.99 | 0.76–1.29 | 0.95 |
| **Behavioural risk environment** | | |  |
| Low risk | Ref | – | – |
| Moderate risk | 1.01 | 0.77–1.28 | 0.97 |
| High risk | 0.82 | 0.62–1.08 | 0.16 |
| **Systolic blood pressure (continuous)** | 1.01 | 1.00–1.01 | 0.12 |
| **Heart rate (continuous)** | 1.00 | 0.99–1.01 | 0.97 |
| **Disability status** | |  |  |
| No disability | Ref | – | – |
| Has disability | 0.9 | 0.64–1.28 | 0.56 |
| Hopelessness (z-score) | 0.9 | 0.76–1.06 | 0.20 |
| Anxiety sensitivity (z-score) | 1.02 | 0.89–1.16 | 0.80 |
| Impulsivity (z-score) | 0.98 | 0.84–1.14 | 0.79 |
| Sensation seeking (z-score) | 1.01 | 0.87–1.15 | 0.98 |
| **Random-effects estimates** | | | |
| **Level** | **Variance (95% CI)** | | |
| Site | 8.55 (2.28–32.08) | | |
| School | 1.95 (1.31–2.91) | | |

**Supplementary Table 12. Multilevel ordinal logistic regression analysis of inhalant use risk among adolescents (N=5,180)**

| **Variable** | **aOR** | **95% CI** | **p-value** |
| --- | --- | --- | --- |
| **Age group** | |  |  |
| 10–13 years | Ref | – | – |
| 14–16 years | 0.72 | 0.56–0.93 | 0.011 |
| 17–19 years | 0.63 | 0.43–0.91 | 0.016 |
| **Gender** |  |  |  |
| Female | Ref | – | – |
| Male | 0.61 | 0.50–0.76 | <0.001 |
| **Household income tertile** | | |  |
| Low income | Ref | – | – |
| Middle income | 1.09 | 0.88–1.35 | 0.44 |
| High income | 1.18 | 0.92–1.51 | 0.19 |
| **Household size** | |  |  |
| ≤5 members | Ref | – | – |
| >5 members | 1.06 | 0.88–1.29 | 0.53 |
| **Father's education** | |  |  |
| Primary or below | Ref | – | – |
| Secondary | 0.90 | 0.72–1.12 | 0.34 |
| Higher education | 1.13 | 0.76–1.69 | 0.54 |
| **Mother's education** | |  |  |
| Primary or below | Ref | – | – |
| Secondary | 1.22 | 0.97–1.53 | 0.08 |
| Higher education | 1.11 | 0.68–1.80 | 0.68 |
| **Religion** |  |  |  |
| Hindu | Ref | – | – |
| Muslim | 0.95 | 0.71–1.27 | 0.72 |
| Others | 0.86 | 0.35–2.15 | 0.75 |
| **School area** | |  |  |
| Rural | Ref | – | – |
| Urban | 1.41 | 0.84–2.36 | 0.19 |
| **School type** | |  |  |
| Government | Ref | – | – |
| Private | 0.68 | 0.33–1.42 | 0.31 |
| **Attitude toward substance use** | | | |
| Low | Ref | – | – |
| Moderate | 1.13 | 0.89–1.43 | 0.31 |
| High | 1.65 | 1.22–2.23 | 0.001 |
| **Accessibility score** |  |  |  |
| Low | Ref | – | – |
| Moderate | 1.92 | 1.54–2.39 | <0.001 |
| High | 1.77 | 1.32–2.39 | <0.001 |
| **Digital exposure** | |  |  |
| Low | Ref | – | – |
| Moderate | 1.28 | 1.02–1.61 | 0.035 |
| High | 1.36 | 1.05–1.76 | 0.018 |
| **Social engagement** | |  |  |
| Low | Ref | – | – |
| Moderate engagement | 0.94 | 0.75–1.19 | 0.60 |
| High engagement | 0.96 | 0.76–1.22 | 0.74 |
| **Behavioural risk environment** | | |  |
| Low risk | Ref | – | – |
| Moderate risk | 1.03 | 0.81–1.30 | 0.81 |
| High risk | 0.95 | 0.73–1.22 | 0.67 |
| **Systolic blood pressure (continuous)** | 1.00 | 1.00–1.01 | 0.15 |
| **Heart rate (continuous)** | 1.00 | 1.00–1.01 | 0.26 |
| **Disability status** | |  |  |
| No disability | Ref | – | – |
| Has disability | 0.95 | 0.69–1.32 | 0.78 |
| Hopelessness (z-score) | 0.82 | 0.70–0.96 | 0.013 |
| Anxiety sensitivity (z-score) | 1.01 | 0.89–1.14 | 0.90 |
| Impulsivity (z-score) | 0.99 | 0.86–1.13 | 0.84 |
| Sensation seeking (z-score) | 1.03 | 0.90–1.17 | 0.66 |
| **Random-effects estimates** | | | |
| **Level** | **Variance (95% CI)** | | |
| Site | 7.45 (1.87–29.67) | | |
| School | 1.64 (1.13–2.38) | | |

**Supplementary Table 13. Multilevel ordinal logistic regression analysis of sedative use risk among adolescents (N=5,180)**

| **Variable** | **aOR** | **95% CI** | **p-value** |
| --- | --- | --- | --- |
| **Age group** | |  |  |
| 10–13 years | Ref | – | – |
| 14–16 years | 0.74 | 0.56–0.98 | 0.033 |
| 17–19 years | 0.56 | 0.37–0.85 | 0.006 |
| **Gender** |  |  |  |
| Female | Ref | – | – |
| Male | 0.67 | 0.53–0.84 | 0.001 |
| **Household income tertile** | | |  |
| Low income | Ref | – | – |
| Middle income | 1.04 | 0.82–1.31 | 0.77 |
| High income | 1.07 | 0.81–1.42 | 0.62 |
| **Household size** | |  |  |
| ≤5 members | Ref | – | – |
| >5 members | 1.01 | 0.81–1.24 | 0.99 |
| **Father's education** | |  |  |
| Primary or below | Ref | – | – |
| Secondary | 1.03 | 0.82–1.30 | 0.80 |
| Higher education | 1.09 | 0.71–1.70 | 0.69 |
| **Mother's education** | |  |  |
| Primary or below | Ref | – | – |
| Secondary | 1.14 | 0.89–1.44 | 0.30 |
| Higher education | 1.33 | 0.79–2.24 | 0.29 |
| **Religion** |  |  |  |
| Hindu | Ref | – | – |
| Muslim | 0.88 | 0.64–1.21 | 0.42 |
| Others | 0.61 | 0.21–1.75 | 0.36 |
| **School area** | |  |  |
| Rural | Ref | – | – |
| Urban | 1.13 | 0.62–2.07 | 0.69 |
| **School type** | |  |  |
| Government | Ref | – | – |
| Private | 0.45 | 0.20–0.99 | 0.048 |
| **Attitude toward substance use** | | | |
| Low | Ref | – | – |
| Moderate | 1.06 | 0.82–1.37 | 0.65 |
| High | 1.81 | 1.29–2.52 | 0.001 |
| **Accessibility score** |  |  |  |
| Low | Ref | – | – |
| Moderate | 2.42 | 1.89–3.10 | <0.001 |
| High | 1.84 | 1.33–2.55 | <0.001 |
| **Digital exposure** | |  |  |
| Low | Ref | – | – |
| Moderate | 1.32 | 1.03–1.71 | 0.03 |
| High | 1.52 | 1.14–2.01 | 0.004 |
| **Social engagement** | |  |  |
| Low | Ref | – | – |
| Moderate engagement | 1.03 | 0.80–1.32 | 0.85 |
| High engagement | 1.06 | 0.81–1.38 | 0.66 |
| **Behavioural risk environment** | | |  |
| Low risk | Ref | – | – |
| Moderate risk | 0.99 | 0.76–1.27 | 0.91 |
| High risk | 0.86 | 0.65–1.14 | 0.29 |
| **Systolic blood pressure (continuous)** | 1.00 | 1.00–1.01 | 0.30 |
| **Heart rate (continuous)** | 1.00 | 1.00–1.01 | 0.46 |
| **Disability status** | |  |  |
| No disability | Ref | – | – |
| Has disability | 1.01 | 0.71–1.40 | 0.99 |
| Hopelessness (z-score) | 0.88 | 0.75–1.03 | 0.12 |
| Anxiety sensitivity (z-score) | 1.02 | 0.89–1.16 | 0.82 |
| Impulsivity (z-score) | 1.06 | 0.91–1.24 | 0.43 |
| Sensation seeking (z-score) | 0.95 | 0.83–1.10 | 0.52 |
| **Random-effects estimates** | | | |
| **Level** | **Variance (95% CI)** | | |
| Site | 8.26 (2.22–30.74) | | |
| School | 2.09 (1.39–3.14) | | |

**Supplementary Table 14. Multilevel ordinal logistic regression analysis of hallucinogen use risk among adolescents (N=5,180)**

| **Variable** | **aOR** | **95% CI** | **p-value** |
| --- | --- | --- | --- |
| **Age group** | |  |  |
| 10–13 years | Ref | – | – |
| 14–16 years | 0.79 | 0.60–1.03 | 0.08 |
| 17–19 years | 0.57 | 0.39–0.85 | 0.006 |
| **Gender** |  |  |  |
| Female | Ref | – | – |
| Male | 0.71 | 0.57–0.89 | 0.002 |
| **Household income tertile** | | |  |
| Low income | Ref | – | – |
| Middle income | 1.03 | 0.82–1.30 | 0.79 |
| High income | 1.23 | 0.94–1.61 | 0.13 |
| **Household size** | |  |  |
| ≤5 members | Ref | – | – |
| >5 members | 0.95 | 0.77–1.16 | 0.60 |
| **Father's education** | |  |  |
| Primary or below | Ref | – | – |
| Secondary | 0.92 | 0.73–1.15 | 0.45 |
| Higher education | 1.04 | 0.68–1.59 | 0.85 |
| **Mother's education** | |  |  |
| Primary or below | Ref | – | – |
| Secondary | 1.17 | 0.93–1.48 | 0.18 |
| Higher education | 1.19 | 0.71–1.99 | 0.50 |
| **Religion** |  |  |  |
| Hindu | Ref | – | – |
| Muslim | 0.78 | 0.57–1.06 | 0.12 |
| Others | 0.70 | 0.26–1.87 | 0.48 |
| **School area** | |  |  |
| Rural | Ref | – | – |
| Urban | 1.11 | 0.60–2.06 | 0.73 |
| **School type** | |  |  |
| Government | Ref | – | – |
| Private | 0.51 | 0.23–1.13 | 0.10 |
| **Attitude toward substance use** | | | |
| Low | Ref | – | – |
| Moderate | 1.07 | 0.83–1.37 | 0.60 |
| High | 1.63 | 1.18–2.25 | 0.003 |
| **Accessibility score** |  |  |  |
| Low | Ref | – | – |
| Moderate | 2.26 | 1.78–2.87 | <0.001 |
| High | 1.79 | 1.31–2.45 | <0.001 |
| **Digital exposure** | |  |  |
| Low | Ref | – | – |
| Moderate | 1.26 | 0.99–1.61 | 0.06 |
| High | 1.37 | 1.04–1.80 | 0.026 |
| **Social engagement** | |  |  |
| Low | Ref | – | – |
| Moderate engagement | 1.02 | 0.80–1.31 | 0.87 |
| High engagement | 0.98 | 0.76–1.26 | 0.85 |
| **Behavioural risk environment** | | |  |
| Low risk | Ref | – | – |
| Moderate risk | 0.99 | 0.78–1.27 | 0.95 |
| High risk | 0.93 | 0.71–1.21 | 0.57 |
| **Systolic blood pressure (continuous)** | 1.00 | 1.00–1.01 | 0.19 |
| **Heart rate (continuous)** | 1.00 | 0.99–1.01 | 0.89 |
| **Disability status** | |  |  |
| No disability | Ref | – | – |
| Has disability | 0.96 | 0.69–1.34 | 0.82 |
| Hopelessness (z-score) | 0.83 | 0.71–0.98 | 0.023 |
| Anxiety sensitivity (z-score) | 1.01 | 0.88–1.15 | 0.92 |
| Impulsivity (z-score) | 1.04 | 0.90–1.20 | 0.57 |
| Sensation seeking (z-score) | 0.97 | 0.85–1.12 | 0.69 |
| **Random-effects estimates** | | | |
| **Level** | **Variance (95% CI)** | | |
| Site | 9.00 (2.44–33.18) | | |
| School | 2.18 (1.43–3.31) | | |

**Supplementary Table 15. Multilevel ordinal logistic regression analysis of opioid use risk among adolescents (N=5,180)**

| **Variable** | **aOR** | **95% CI** | **p-value** |
| --- | --- | --- | --- |
| **Age group** | |  |  |
| 10–13 years | Ref | – | – |
| 14–16 years | 0.78 | 0.59–1.03 | 0.08 |
| 17–19 years | 0.57 | 0.38–0.87 | 0.008 |
| **Gender** |  |  |  |
| Female | Ref | – | – |
| Male | 0.69 | 0.55–0.87 | 0.001 |
| **Household income tertile** | | |  |
| Low income | Ref | – | – |
| Middle income | 1.13 | 0.89–1.43 | 0.33 |
| High income | 1.22 | 0.92–1.61 | 0.17 |
| **Household size** | |  |  |
| ≤5 members | Ref | – | – |
| >5 members | 0.96 | 0.77–1.19 | 0.70 |
| **Father's education** | |  |  |
| Primary or below | Ref | – | – |
| Secondary | 0.98 | 0.77–1.24 | 0.86 |
| Higher education | 1.11 | 0.72–1.71 | 0.65 |
| **Mother's education** | |  |  |
| Primary or below | Ref | – | – |
| Secondary | 1.08 | 0.85–1.37 | 0.53 |
| Higher education | 1.05 | 0.62–1.78 | 0.85 |
| **Religion** |  |  |  |
| Hindu | Ref | – | – |
| Muslim | 0.90 | 0.66–1.24 | 0.52 |
| Others | 0.65 | 0.23–1.87 | 0.43 |
| **School area** | |  |  |
| Rural | Ref | – | – |
| Urban | 1.27 | 0.68–2.35 | 0.45 |
| **School type** | |  |  |
| Government | Ref | – | – |
| Private | 0.56 | 0.26–1.22 | 0.14 |
| **Attitude toward substance use** | | | |
| Low | Ref | – | – |
| Moderate | 1.18 | 0.91–1.53 | 0.21 |
| High | 2.08 | 1.48–2.91 | <0.001 |
| **Accessibility score** |  |  |  |
| Low | Ref | – | – |
| Moderate | 2.20 | 1.71–2.82 | <0.001 |
| High | 1.85 | 1.33–2.56 | <0.001 |
| **Digital exposure** | |  |  |
| Low | Ref | – | – |
| Moderate | 1.34 | 1.04–1.73 | 0.024 |
| High | 1.43 | 1.07–1.91 | 0.014 |
| **Social engagement** | |  |  |
| Low | Ref | – | – |
| Moderate engagement | 1.01 | 0.78–1.31 | 0.94 |
| High engagement | 1.02 | 0.78–1.33 | 0.90 |
| **Behavioural risk environment** | | |  |
| Low risk | Ref | – | – |
| Moderate risk | 0.92 | 0.72–1.19 | 0.55 |
| High risk | 0.84 | 0.64–1.11 | 0.22 |
| **Systolic blood pressure (continuous)** | 1.00 | 1.00–1.01 | 0.24 |
| **Heart rate (continuous)** | 1.00 | 0.99–1.01 | 0.99 |
| **Disability status** | |  |  |
| No disability | Ref | – | – |
| Has disability | 1.05 | 0.75–1.48 | 0.78 |
| Hopelessness (z-score) | 0.92 | 0.78–1.08 | 0.30 |
| Anxiety sensitivity (z-score) | 0.98 | 0.86–1.13 | 0.82 |
| Impulsivity (z-score) | 1.08 | 0.93–1.26 | 0.32 |
| Sensation seeking (z-score) | 0.94 | 0.82–1.09 | 0.41 |
| **Random-effects estimates** | | | |
| **Level** | **Variance (95% CI)** | | |
| Site | 12.47 (3.32–46.81) | | |
| School | 2.01 (1.32–3.06) | | |

**Supplementary Table 16. Cross-substance summary of statistically significant predictors from multilevel ordinal logistic regression models**

| **Variable** | **Tobacco** | **Alcohol** | **Cannabis** | **Cocaine** | **Amphetamine** | **Inhalant** | **Sedative** | **Hallucinogen** | **Opioid** |
| --- | --- | --- | --- | --- | --- | --- | --- | --- | --- |
| **Age group** |  |  |  |  |  |  |  |  |  |
| 10–13 years | Ref | Ref | Ref | Ref | Ref | Ref | Ref | Ref | Ref |
| 14–16 years | 0.79† | 0.69* | 0.78† | 0.73* | 0.77† | 0.72* | 0.74* | 0.79† | 0.78† |
| 17–19 years | 0.63* | 0.52** | 0.61* | 0.60* | 0.55** | 0.63* | 0.56** | 0.57** | 0.57** |
| **Gender** |  |  |  |  |  |  |  |  |  |
| Female | Ref | Ref | Ref | Ref | Ref | Ref | Ref | Ref | Ref |
| Male | 0.75** | 0.62*** | 0.63*** | 0.68*** | 0.66*** | 0.61*** | 0.67*** | 0.71** | 0.69** |
| **Income tertile** |  |  |  |  |  |  |  |  |  |
| Low income | Ref | Ref | Ref | Ref | Ref | Ref | Ref | Ref | Ref |
| Middle income | 1.12 | 1.15 | 1.09 | 1.1 | 1.05 | 1.09 | 1.04 | 1.03 | 1.13 |
| High income | 1.09 | 1.48* | 1.23 | 1.15 | 1.05 | 1.18 | 1.07 | 1.23 | 1.22 |
| **School type** |  |  |  |  |  |  |  |  |  |
| Government | Ref | Ref | Ref | Ref | Ref | Ref | Ref | Ref | Ref |
| Private | 0.83 | 0.49† | 0.63 | 0.55 | 0.50† | 0.68 | 0.45* | 0.51† | 0.56 |
| **Attitude score** |  |  |  |  |  |  |  |  |  |
| Low | Ref | Ref | Ref | Ref | Ref | Ref | Ref | Ref | Ref |
| Moderate | 0.89 | 1.24 | 0.98 | 0.96 | 1.06 | 1.13 | 1.06 | 1.07 | 1.18 |
| High | 1.33† | 2.15*** | 1.58** | 1.50* | 1.93*** | 1.65** | 1.81*** | 1.63** | 2.08*** |
| **Accessibility score** |  |  |  |  |  |  |  |  |  |
| Low | Ref | Ref | Ref | Ref | Ref | Ref | Ref | Ref | Ref |
| Moderate | 2.28*** | 2.57*** | 2.53*** | 2.26*** | 2.46*** | 1.92*** | 2.42*** | 2.26*** | 2.20*** |
| High | 2.24*** | 3.07*** | 2.42*** | 1.91*** | 2.01*** | 1.77*** | 1.84*** | 1.79*** | 1.85*** |
| **Digital exposure** |  |  |  |  |  |  |  |  |  |
| Low | Ref | Ref | Ref | Ref | Ref | Ref | Ref | Ref | Ref |
| Moderate | 1.47** | 1.46* | 1.25† | 1.26† | 1.38* | 1.28* | 1.32* | 1.26† | 1.34* |
| High | 1.45** | 1.67** | 1.38* | 1.42* | 1.44* | 1.36* | 1.52** | 1.37* | 1.43* |
| Hopelessness (z-score) | 0.81** | 0.82* | 0.85† | 0.87 | 0.90 | 0.82* | 0.88 | 0.83* | 0.92 |

Note: Estimates are adjusted odds ratios (aORs) derived from multilevel ordinal logistic regression models with random intercepts for study site and school. All models were adjusted for age group, gender, household income tertile, household size, father's education, mother's education, religion, school location (urban/rural), school type, substance-related attitude, accessibility, digital exposure, social engagement, behavioural risk environment, systolic blood pressure, heart rate, disability status, hopelessness, anxiety sensitivity, impulsivity, and sensation-seeking personality traits. Only selected predictors are displayed for brevity; full model estimates are provided in Supplementary Tables S7–S15. Statistical significance is denoted as * p<0.05; ** p<0.01; *** p<0.001; † p<0.10.

**Supplementary Table 17: Summary of Inter-site Difference in Moderate/High Substance Use (Guwahati vs Nagpur)**

| **Measure** | **Value** |
| --- | --- |
| Total sample included in decomposition | 1,972 |
| Adolescents from Nagpur | 1,082 |
| Adolescents from Guwahati | 890 |
| Prevalence of moderate/high substance use risk in Nagpur, Pr (Y≠0 \| G=0) | 0.071 (7.1%) |
| Prevalence of moderate/high substance use risk in Guwahati, Pr (Y≠0 \| G=1) | 0.422 (42.2%) |
| Absolute difference in prevalence | 0.351 (35.1% points) |
| Explained difference | 0.133 |
| Percentage of difference explained | 37.9% |

**Supplementary file 18: SUBSTANCE USE FREQUENCY – WHO ASSIST**

**अनुभाग F: नशीले पदार्थों के उपयोग की आवृत्ति – WHO ASSIST**

**F1. In your life, which of the following substances have you ever used?**

**F1. अपने जीवन में, क्या आपने निम्नलिखित में से किसी नशीले पदार्थ का उपयोग कभी किया है?**

| **S.No.**  **क्रमांक** | **Substance** | **पदार्थ** | **No (नहीं) [0]** | **Yes (हाँ) [3]** |
| --- | --- | --- | --- | --- |
| **a)** | **Tobacco products (cigarettes, chewing tobacco, cigars, etc.)** | **तंबाकू उत्पाद (सिगरेट, चबाने वाला तंबाकू, सिगार, सूर्ति, खैनी आदि)** | **☐** | **☐** |
| **b)** | **Alcoholic beverages (beer, wine, spirits, etc.)** | **मादक पेय पदार्थ (बीयर, वाइन, शराब, ताड़ी आदि)** | **☐** | **☐** |
| **c)** | **Cannabis (marijuana, pot, grass, hash, etc.)** | **गांजा (मारिजुआना, हशीश आदि)** | **☐** | **☐** |
| **d)** | **Cocaine** | **कोकीन** | **☐** | **☐** |
| **e)** | **Amphetamine-type stimulants (speed, diet pills, ecstasy, etc.)** | **एम्फ़ैटेमीन प्रकार उत्तेजक (स्पीड, डाइट पिल्स, एक्स्टसी आदि)** | **☐** | **☐** |
| **f)** | **Inhalants (nitrous, glue, petrol, paint thinner, etc.)** | **इनहेलेंट्स (नाइट्रस, गोंद, पेट्रोल, पेंट थिनर, व्हिटनर, डेंडराइट, सॉल्यूशन आदि)** | **☐** | **☐** |
| **g)** | **Sedatives or Sleeping Pills (Valium, Serepax, Rohypnol, etc.)** | **शांतिकारक या नींद की गोलियां (वैलियम, सेरेपैक्स, रोहिपनॉल आदि)** | **☐** | **☐** |
| **h)** | **Hallucinogens (LSD, acid, PCP,Special K, etc.)** | **(एलएसडी, एसिड, पीसीपी, भांग, जड़ी-बूटी का काढ़ा आदि)** | **☐** | **☐** |
| **i)** | **Opioids (heroin, morphine, methadone, codeine, etc.)** | **ओपिओइड्स (हेरोइन, मॉर्फिन, मेथाडोन, कोडीन आदि)** | **☐** | **☐** |
| **j)** | **Other – specify** | **अन्य – स्ष्ट करें** | **☐** | **☐** |
| **If "No" to all items, stop interview. If "Yes" to any of these items, ask Question 2 for each substance ever used.”**  **यदि सभी विकल्पों के लिए ‘नहीं’ का उत्तर दिया गया है, तो साक्षात्कार यहीं समाप्त करें। यदि किसी भी विकल्प के लिए ‘हाँ’ का उत्तर दिया गया है, तो उपयोग किए गए प्रत्येक पदार्थ के लिए प्रश्न 2 पूछें।”** | | | | |

**Question F2: In the past three months, how often have you used the substances you mentioned? “पिछले 3 महीनों में आपने जिन पदार्थों का उपयोग किया है, उन्हें कितनी बार इस्तेमाल किया है?**

| **S.No.**  **क्रमांक** | **Substance** | **पदार्थ** | **Never**  **(कभी नहीं) [0]** | **Once or twice**  **(एक या दो बार) [2]** | **Monthly (मासिक) [3]** | **Weekly (साप्ताहिक) [4]** | **Daily or almost daily (रोज़ाना या लगभग रोज़) [6]** |
| --- | --- | --- | --- | --- | --- | --- | --- |
| **a)** | **Tobacco products (cigarettes, chewing tobacco, cigars, etc.)** | **तंबाकू उत्पाद (सिगरेट, चबाने वाला तंबाकू, सिगार, सूर्ति, खैनी आदि)** | **☐** | **☐** | **☐** | **☐** | **☐** |
| **b)** | **Alcoholic beverages (beer, wine, spirits, etc.)** | **मादक पेय पदार्थ (बीयर, वाइन, शराब, ताड़ी आदि)** | **☐** | **☐** | **☐** | **☐** | **☐** |
| **c)** | **Cannabis (marijuana, pot, grass, hash, etc.)** | **गांजा (मारिजुआना, हशीश आदि)** | **☐** | **☐** | **☐** | **☐** | **☐** |
| **d)** | **Cocaine** | **कोकीन** | **☐** | **☐** | **☐** | **☐** | **☐** |
| **e)** | **Amphetamine-type stimulants (speed, diet pills, ecstasy, etc.)** | **एम्फ़ैटेमीन प्रकार उत्तेजक (स्पीड, डाइट पिल्स, एक्स्टसी आदि)** | **☐** | **☐** | **☐** | **☐** | **☐** |
| **f)** | **Inhalants (nitrous, glue, petrol, paint thinner, etc.)** | **इनहेलेंट्स (नाइट्रस, गोंद, पेट्रोल, पेंट थिनर, व्हिटनर, डेंडराइट, सॉल्यूशन आदि)** | **☐** | **☐** | **☐** | **☐** | **☐** |
| **g)** | **Sedatives or Sleeping Pills (Valium, Serepax, Rohypnol, etc.)** | **शांतिकारक या नींद की गोलियां (वैलियम, सेरेपैक्स, रोहिपनॉल आदि)** | **☐** | **☐** | **☐** | **☐** | **☐** |
| **h)** | **Hallucinogens (LSD, acid, PCP, Special K, etc.)** | **(एलएसडी, एसिड, पीसीपी, भांग, जड़ी-बूटी का काढ़ा आदि)** | **☐** | **☐** | **☐** | **☐** | **☐** |
| **i)** | **Opioids (heroin, morphine, methadone, codeine, etc.)** | **ओपिओइड्स (हेरोइन, मॉर्फिन, मेथाडोन, कोडीन आदि)** | **☐** | **☐** | **☐** | **☐** | **☐** |
| **j)** | **Other – specify** | **अन्य – स्ष्ट करें** | **☐** | **☐** | **☐** | **☐** | **☐** |
| **If "Never" to all items in Question 2, skip to Question 6. If any substances in Question 2 were used in the previous three months, continue with Questions 3, 4 & 5 for e ach substance used.**  **“यदि प्रश्न 2 के सभी विकल्पों के लिए ‘कभी नहीं’ का उत्तर दिया गया है, तो सीधे प्रश्न 6 पर जाएं। यदि प्रश्न 2 में किसी भी पदार्थ का उपयोग पिछले तीन महीनों में किया गया है, तो उपयोग किए गए प्रत्येक पदार्थ के लिए प्रश्न 3, 4 और 5 पूछें।”** | | | | | | | |

**Question F3: During the past three months, how often have you had a strong desire or urge to use? पिछले तीन महीनों में, आपको किसी नशीले पदार्थ का उपयोग करने की तीव्र इच्छा या मजबूरी कितनी बार महसूस हुई?**

| **S.No. क्रमांक** | **Substance** | **पदार्थ** | **Never**  **(कभी नहीं)**  **[0]** | **Once or twice**  **(एक या दो बार) [3]** | **Monthly (मासिक) [4]** | **Weekly (साप्ताहिक) [5]** | **Daily or almost daily (रोज़ाना या लगभग रोज़) [6]** |
| --- | --- | --- | --- | --- | --- | --- | --- |
| **a)** | **Tobacco products (cigarettes, chewing tobacco, cigars, etc.)** | **तंबाकू उत्पाद (सिगरेट, चबाने वाला तंबाकू, सिगार, सूर्ति, खैनी आदि)** | **☐** | **☐** | **☐** | **☐** | **☐** |
| **b)** | **Alcoholic beverages (beer, wine, spirits, etc.)** | **मादक पेय पदार्थ (बीयर, वाइन, शराब, ताड़ी आदि)** | **☐** | **☐** | **☐** | **☐** | **☐** |
| **c)** | **Cannabis (marijuana, pot, grass, hash, etc.)** | **गांजा (मारिजुआना, हशीश आदि)** | **☐** | **☐** | **☐** | **☐** | **☐** |
| **d)** | **Cocaine** | **कोकीन** | **☐** | **☐** | **☐** | **☐** | **☐** |
| **e)** | **Amphetamine-type stimulants (speed, diet pills, ecstasy, etc.)** | **एम्फ़ैटेमीन प्रकार उत्तेजक (स्पीड, डाइट पिल्स, एक्स्टसी आदि)** | **☐** | **☐** | **☐** | **☐** | **☐** |
| **f)** | **Inhalants (nitrous, glue, petrol, paint thinner, etc.)** | **इनहेलेंट्स (नाइट्रस, गोंद, पेट्रोल, पेंट थिनर, व्हिटनर, डेंडराइट, सॉल्यूशन आदि)** | **☐** | **☐** | **☐** | **☐** | **☐** |
| **g)** | **Sedatives or Sleeping Pills (Valium, Serepax, Rohypnol, etc.)** | **शांतिकारक या नींद की गोलियां (वैलियम, सेरेपैक्स, रोहिपनॉल आदि)** | **☐** | **☐** | **☐** | **☐** | **☐** |
| **h)** | **Hallucinogens (LSD, acid, PCP, Special K, etc.)** | **(एलएसडी, एसिड, पीसीपी, भांग, जड़ी-बूटी का काढ़ा आदि)** | **☐** | **☐** | **☐** | **☐** | **☐** |
| **i)** | **Opioids (heroin, morphine, methadone, codeine, etc.)** | **ओपिओइड्स (हेरोइन, मॉर्फिन, मेथाडोन, कोडीन आदि)** | **☐** | **☐** | **☐** | **☐** | **☐** |
| **j)** | **Other – specify** | **अन्य – स्ष्ट करें** | **☐** | **☐** | **☐** | **☐** | **☐** |

**Question F4: During the past three months, how often has your use of [substance] led to health, social, legal or financial problems? “पिछले 3 महीने में [नशे] की वजह से क्या आपको सेहत, घर-परिवार, कानून या पैसे की परेशानी कितनी बार हुई?**

| **S.No.** | **Substance** | **पदार्थ** | **Never (कभी नहीं) [0]** | **Once or twice (एक या दो बार) [4]** | **Monthly (मासिक) [5]** | **Weekly (साप्ताहिक) [6]** | **Daily or almost daily (रोज़ाना या लगभग रोज़) [7]** |
| --- | --- | --- | --- | --- | --- | --- | --- |
| **a)** | **Tobacco products (cigarettes, chewing tobacco, cigars, etc.)** | **तंबाकू उत्पाद (सिगरेट, चबाने वाला तंबाकू, सिगार, सूर्ति, खैनी आदि)** | **☐** | **☐** | **☐** | **☐** | **☐** |
| **b)** | **Alcoholic beverages (beer, wine, spirits, etc.)** | **मादक पेय पदार्थ (बीयर, वाइन, शराब, ताड़ी आदि)** | **☐** | **☐** | **☐** | **☐** | **☐** |
| **c)** | **Cannabis (marijuana, pot, grass, hash, etc.)** | **गांजा (मारिजुआना, हशीश आदि)** | **☐** | **☐** | **☐** | **☐** | **☐** |
| **d)** | **Cocaine (coke, crack, etc.)** | **कोकीन** | **☐** | **☐** | **☐** | **☐** | **☐** |
| **e)** | **Amphetamine-type stimulants (speed, diet pills, ecstasy, etc.)** | **एम्फ़ैटेमीन प्रकार उत्तेजक (स्पीड, डाइट पिल्स, एक्स्टसी आदि)** | **☐** | **☐** | **☐** | **☐** | **☐** |
| **f)** | **Inhalants (nitrous, glue, petrol, paint thinner, etc.)** | **इनहेलेंट्स (नाइट्रस, गोंद, पेट्रोल, पेंट थिनर, व्हिटनर, डेंडराइट, सॉल्यूशन आदि)** | **☐** | **☐** | **☐** | **☐** | **☐** |
| **g)** | **Sedatives or Sleeping Pills (Valium, Serepax, Rohypnol, etc.)** | **शांतिकारक या नींद की गोलियां (वैलियम, सेरेपैक्स, रोहिपनॉल आदि)** | **☐** | **☐** | **☐** | **☐** | **☐** |
| **h)** | **Hallucinogens (LSD, acid, PCP, Special K, etc.)** | **(एलएसडी, एसिड, पीसीपी, भांग, जड़ी-बूटी का काढ़ा आदि)** | **☐** | **☐** | **☐** | **☐** | **☐** |
| **i)** | **Opioids (heroin, morphine, methadone, codeine, etc.)** | **ओपिओइड्स (हेरोइन, मॉर्फिन, मेथाडोन, कोडीन आदि)** | **☐** | **☐** | **☐** | **☐** | **☐** |
| **j)** | **Other – specify** | **अन्य – स्ष्ट करें** | **☐** | **☐** | **☐** | **☐** | **☐** |

**Question F5: During the past three months, how often have you failed to do what was normally expected of you because of your use of [substance** **पिछले 3 महीने में [नशे] की वजह से आपसे जो रोजमर्रा के काम या जिम्मेदारी की उम्मीद थी, वो कितनी बार नहीं हो पाई?**

| **S.No. क्रमांक** | **Substance** | **पदार्थ** | **Never (कभी नहीं) [0]** | **Once or twice (एक या दो बार) [5]** | **Monthly (मासिक) [6]** | **Weekly (साप्ताहिक) [7]** | **Daily or almost daily (रोज़ाना या लगभग रोज़) [8]** |
| --- | --- | --- | --- | --- | --- | --- | --- |
| **a)** | **Tobacco products (cigarettes, chewing tobacco, cigars, etc.)** | **तंबाकू उत्पाद (सिगरेट, चबाने वाला तंबाकू, सिगार, सूर्ति, खैनी आदि)** | **☐** | **☐** | **☐** | **☐** | **☐** |
| **b)** | **Alcoholic beverages (beer, wine, spirits, etc.)** | **मादक पेय पदार्थ (बीयर, वाइन, शराब, ताड़ी आदि)** | **☐** | **☐** | **☐** | **☐** | **☐** |
| **c)** | **Cannabis (marijuana, pot, grass, hash, etc.)** | **गांजा (मारिजुआना, हशीश आदि)** | **☐** | **☐** | **☐** | **☐** | **☐** |
| **d)** | **Cocaine (coke, crack, etc.)** | **कोकीन** | **☐** | **☐** | **☐** | **☐** | **☐** |
| **e)** | **Amphetamine-type stimulants (speed, diet pills, ecstasy, etc.)** | **एम्फ़ैटेमीन प्रकार उत्तेजक (स्पीड, डाइट पिल्स, एक्स्टसी आदि)** | **☐** | **☐** | **☐** | **☐** | **☐** |
| **f)** | **Inhalants (nitrous, glue, petrol, paint thinner, etc.)** | **इनहेलेंट्स (नाइट्रस, गोंद, पेट्रोल, पेंट थिनर, व्हिटनर, डेंडराइट, सॉल्यूशन आदि)** | **☐** | **☐** | **☐** | **☐** | **☐** |
| **g)** | **Sedatives or Sleeping Pills (Valium, Serepax, Rohypnol, etc.)** | **शांतिकारक या नींद की गोलियां (वैलियम, सेरेपैक्स, रोहिपनॉल आदि)** | **☐** | **☐** | **☐** | **☐** | **☐** |
| **h)** | **Hallucinogens (LSD, acid, PCP, Special K, etc.)** | **(एलएसडी, एसिड, पीसीपी, भांग, जड़ी-बूटी का काढ़ा आदि)** | **☐** | **☐** | **☐** | **☐** | **☐** |
| **i)** | **Opioids (heroin, morphine, methadone, codeine, etc.)** | **ओपिओइड्स (हेरोइन, मॉर्फिन, मेथाडोन, कोडीन आदि)** | **☐** | **☐** | **☐** | **☐** | **☐** |
| **j)** | **Other – specify** | **अन्य – स्ष्ट करें** | **☐** | **☐** | **☐** | **☐** | **☐** |

**Question F6: Has a friend or relative or anyone else ever expressed concern about your use of [substance]? क्या किसी दोस्त, रिश्तेदार या किसी और ने कभी आपके [पदार्थ] के उपयोग को लेकर चिंता जताई है?**

| **S.No.**  **क्रमांक** | **Substance** | **पदार्थ** | **No, Never  (नहीं, कभी नहीं) [0]** | **Yes, in the past three months (हाँ, पिछले तीन महीनों में) [6]** | **Yes, but not in past three months (हाँ, लेकिन पिछले तीन महीनों में नहीं) [3]** |
| --- | --- | --- | --- | --- | --- |
| **a)** | **Tobacco products (cigarettes, chewing tobacco, cigars, etc.)** | **तंबाकू उत्पाद (सिगरेट, चबाने वाला तंबाकू, सिगार, सूर्ति, खैनी आदि)** | **☐** | **☐** | **☐** |
| **b)** | **Alcoholic beverages (beer, wine, spirits, etc.)** | **मादक पेय पदार्थ (बीयर, वाइन, शराब, ताड़ी आदि)** | **☐** | **☐** | **☐** |
| **c)** | **Cannabis (marijuana, pot, grass, hash, etc.)** | **गांजा (मारिजुआना, हशीश आदि)** | **☐** | **☐** | **☐** |
| **d)** | **Cocaine (coke, crack, etc.)** | **कोकीन** | **☐** | **☐** | **☐** |
| **e)** | **Amphetamine-type stimulants (speed, diet pills, ecstasy, etc.)** | **एम्फ़ैटेमीन प्रकार उत्तेजक (स्पीड, डाइट पिल्स, एक्स्टसी आदि)** | **☐** | **☐** | **☐** |
| **f)** | **Inhalants (nitrous, glue, petrol, paint thinner, etc.)** | **इनहेलेंट्स (नाइट्रस, गोंद, पेट्रोल, पेंट थिनर, व्हिटनर, डेंडराइट, सॉल्यूशन आदि)** | **☐** | **☐** | **☐** |
| **g)** | **Sedatives or Sleeping Pills (Valium, Serepax, Rohypnol, etc.)** | **शांतिकारक या नींद की गोलियां (वैलियम, सेरेपैक्स, रोहिपनॉल आदि)** | **☐** | **☐** | **☐** |
| **h)** | **Hallucinogens (LSD, acid, PCP, Special K, etc.)** | **(एलएसडी, एसिड, पीसीपी, भांग, जड़ी-बूटी का काढ़ा आदि)** | **☐** | **☐** | **☐** |
| **i)** | **Opioids (heroin, morphine, methadone, codeine, etc.)** | **ओपिओइड्स (हेरोइन, मॉर्फिन, मेथाडोन, कोडीन आदि)** | **☐** | **☐** | **☐** |
| **j)** | **Other – specify** | **अन्य – स्ष्ट करें** | **☐** | **☐** | **☐** |

**Question F7: Have you ever tried and failed to control, cut down or stop using [substance]? क्या आपने कभी [पदार्थ] का उपयोग नियंत्रित करने, कम करने या रोकने की कोशिश की है और असफल रहे हैं?**

| **S.No.** | **Substance** | **पदार्थ** | **No, Never (नहीं, कभी नहीं) [0]** | **Yes, in the past three months (हाँ, पिछले तीन महीनों में) [6]** | **Yes, but not in past three months (हाँ, लेकिन पिछले तीन महीनों में नहीं) [3]** |
| --- | --- | --- | --- | --- | --- |
| **a)** | **Tobacco products (cigarettes, chewing tobacco, cigars, etc.)** | **तंबाकू उत्पाद (सिगरेट, चबाने वाला तंबाकू, सिगार, सूर्ति, खैनी आदि)** | **☐** | **☐** | **☐** |
| **b)** | **Alcoholic beverages (beer, wine, spirits, etc.)** | **मादक पेय पदार्थ (बीयर, वाइन, शराब, ताड़ी आदि)** | **☐** | **☐** | **☐** |
| **c)** | **Cannabis (marijuana, pot, grass, hash, etc.)** | **गांजा (मारिजुआना, हशीश आदि)** | **☐** | **☐** | **☐** |
| **d)** | **Cocaine (coke, crack, etc.)** | **कोकीन** | **☐** | **☐** | **☐** |
| **e)** | **Amphetamine-type stimulants (speed, diet pills, ecstasy, etc.)** | **एम्फ़ैटेमीन प्रकार उत्तेजक (स्पीड, डाइट पिल्स, एक्स्टसी आदि)** | **☐** | **☐** | **☐** |
| **f)** | **Inhalants (nitrous, glue, petrol, paint thinner, etc.)** | **इनहेलेंट्स (नाइट्रस, गोंद, पेट्रोल, पेंट थिनर, व्हिटनर, डेंडराइट, सॉल्यूशन आदि)** | **☐** | **☐** | **☐** |
| **g)** | **Sedatives or Sleeping Pills (Valium, Serepax, Rohypnol, etc.)** | **शांतिकारक या नींद की गोलियां (वैलियम, सेरेपैक्स, रोहिपनॉल आदि)** | **☐** | **☐** | **☐** |
| **h)** | **Hallucinogens (LSD, acid, PCP, Special K, etc.)** | **(एलएसडी, एसिड, पीसीपी, भांग, जड़ी-बूटी का काढ़ा आदि)** | **☐** | **☐** | **☐** |
| **i)** | **Opioids (heroin, morphine, methadone, codeine, etc.)** | **ओपिओइड्स (हेरोइन, मॉर्फिन, मेथाडोन, कोडीन आदि)** | **☐** | **☐** | **☐** |
| **j)** | **Other – specify** | **अन्य – स्ष्ट करें** | **☐** | **☐** | **☐** |

**Calculation of ASSIST Substance-Specific Involvement Scores**

For each substance category, the Specific Substance Involvement Score (SSIS) was calculated by summing the scores from Questions 2 to 7 of the WHO-ASSIST Version 3.1. Responses to Question 1 (lifetime use) was not included in the score calculation.

For example, the cannabis-specific score was calculated as:

**Cannabis score = F2c + F3c + F4c + F5c + F6c + F7c**

For tobacco, Question 5 is not applicable and is therefore excluded:

**Tobacco score = F2a + F3a + F4a + F6a + F7a**

**Interpretation of ASSIST Substance-Specific Involvement Scores**

| **Substance** | **Low Risk** | **Moderate Risk** | **High Risk** |
| --- | --- | --- | --- |
| Tobacco | 0–3 | 4–26 | ≥27 |
| Alcohol | 0–10 | 11–26 | ≥27 |
| Cannabis | 0–3 | 4–26 | ≥27 |
| Cocaine | 0–3 | 4–26 | ≥27 |
| Amphetamine-type stimulants | 0–3 | 4–26 | ≥27 |
| Inhalants | 0–3 | 4–26 | ≥27 |
| Sedatives or sleeping pills | 0–3 | 4–26 | ≥27 |
| Hallucinogens | 0–3 | 4–26 | ≥27 |
| Opioids | 0–3 | 4–26 | ≥27 |
| Other drugs | 0–3 | 4–26 | ≥27 |

**Supplementary Table 19. Sensitivity analysis of determinants of substance use risk among school-going adolescents in India using a three-level multilevel ordinal logistic regression model excluding accessibility of substances as a covariate (N = 6,168)**

| **Variable** | **Adjusted OR** | **95% CI** | **p-value** |
| --- | --- | --- | --- |
| **Age group** |  |  |  |
| 10–13 years | Ref |  |  |
| 14–16 years | 0.85 | 0.68–1.07 | 0.17 |
| 17–19 years | 0.77 | 0.56–1.06 | 0.11 |
| **Gender** |  |  |  |
| Female | Ref |  |  |
| Male | 0.74 | 0.62–0.88 | 0.001 |
| **Monthly family income** |  |  |  |
| Low income | Ref |  |  |
| Middle income | 1.12 | 0.93–1.34 | 0.22 |
| High income | 1.08 | 0.88–1.32 | 0.49 |
| **Household size** |  |  |  |
| ≤5 members | Ref |  |  |
| >5 members | 1.05 | 0.89–1.24 | 0.59 |
| **Father's education** |  |  |  |
| Low | Ref |  |  |
| Secondary | 0.92 | 0.76–1.10 | 0.35 |
| Higher | 0.94 | 0.67–1.33 | 0.73 |
| **Mother's education** |  |  |  |
| Low | Ref |  |  |
| Secondary | 1.15 | 0.95–1.38 | 0.16 |
| Higher | 1.01 | 0.66–1.52 | 0.99 |
| **Religion** |  |  |  |
| Hindu | Ref |  |  |
| Muslim | 0.89 | 0.70–1.13 | 0.32 |
| Others | 0.90 | 0.43–1.88 | 0.78 |
| **Area of school** |  |  |  |
| Rural | Ref |  |  |
| Urban | 1.07 | 0.69–1.66 | 0.77 |
| **School type** |  |  |  |
| Government | Ref |  |  |
| Private | 0.97 | 0.52–1.81 | 0.92 |
| **Attitude toward substance use** |  |  |  |
| Low | Ref |  |  |
| Moderate | 0.98 | 0.80–1.19 | 0.81 |
| High | 1.27 | 0.98–1.64 | 0.07 |
| **Digital exposure** |  |  |  |
| Low | Ref |  |  |
| Moderate | 1.38 | 1.13–1.68 | 0.002 |
| High | 1.56 | 1.26–1.94 | <0.001 |
| **Social engagement** |  |  |  |
| Low | Ref |  |  |
| Moderate | 0.92 | 0.75–1.12 | 0.40 |
| High | 0.97 | 0.79–1.18 | 0.76 |
| **Risky behavioural environment** |  |  |  |
| Low | Ref |  |  |
| Moderate | 1.06 | 0.87–1.30 | 0.54 |
| High | 1.07 | 0.86–1.32 | 0.54 |
| Systolic blood pressure | 1.00 | 1.00–1.01 | 0.10 |
| Heart rate | 1.00 | 1.00–1.01 | 0.63 |
| Disability | 1.05 | 0.80–1.39 | 0.71 |
| **Personality Traits (SURPS domains)** |  |  |  |
| Hopelessness (z-score) | 0.90 | 0.79–1.02 | 0.11 |
| Anxiety sensitivity (z-score) | 0.98 | 0.88–1.08 | 0.64 |
| Impulsivity (z-score) | 1.02 | 0.91–1.14 | 0.77 |
| Sensation seeking (z-score) | 1.02 | 0.91–1.14 | 0.77 |
| **Random-effects parameters** | **Variance** | **SE** | **95% CI** |
| **Study-site level** (random intercept) | 5.71 | 3.61 | 1.66–19.69 |
| **School level** (random intercept) | 1.29 | 0.24 | 0.90–1.85 |

Note: To assess the robustness of the primary findings, a sensitivity analysis was conducted by excluding the accessibility of substances variable from the multilevel ordinal logistic regression model. Accessibility-related information was unavailable for all participants from the Puducherry study site (n = 988), which reduced the analytic sample in the primary complete-case analysis. Excluding accessibility from the model allowed inclusion of the full study sample (N = 6,168), including Puducherry. Results from this sensitivity analysis were compared with those from the primary model to evaluate the influence of missing accessibility data on the observed associations. The direction and overall pattern of associations remained broadly similar, supporting the robustness of the primary findings. Models included random intercepts at both the study-site and school levels. Adjusted odds ratios (aORs), 95% confidence intervals (CIs), and p-values are presented.


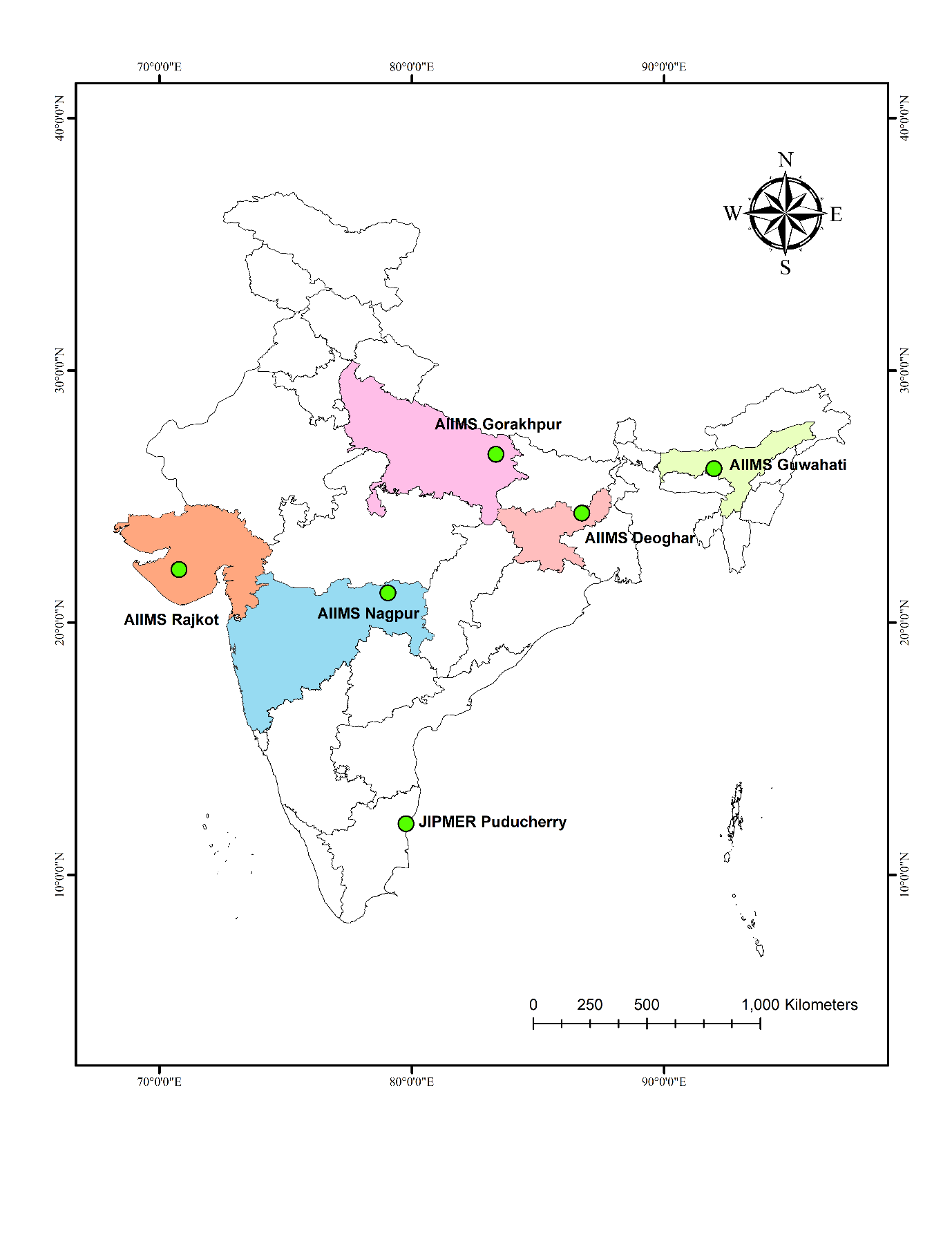


**Supplementary Figure 1:** **Distribution of the six study sites included in the adolescent substance use survey in India (2025–2026)**
